# Optimizing Treatment Regimes to Hinder Antiviral Resistance in Influenza across Time Scales

**DOI:** 10.1371/journal.pone.0059529

**Published:** 2013-03-29

**Authors:** Oscar Patterson-Lomba, Benjamin M. Althouse, Georg M. Goerg, Laurent Hébert-Dufresne

**Affiliations:** 1 Mathematical, Computational, and Modeling Sciences Center, School of Human Evolution and Social Change, Arizona State University, Tempe, Arizona, United States of America; 2 Department of Epidemiology, Johns Hopkins Bloomberg School of Public Health, Baltimore, Maryland, United States of America; 3 Department of Statistics, Carnegie Mellon University, Pittsburgh, Pennsylvania, United States of America; 4 Département de Physique, de Génie Physique, et d’Optique, Université Laval, Québec, Québec, Canada; Harvard School of Public Health, United States of America

## Abstract

The large-scale use of antivirals during influenza pandemics poses a significant selection pressure for drug-resistant pathogens to emerge and spread in a population. This requires treatment strategies to minimize total infections as well as the emergence of resistance. Here we propose a mathematical model in which individuals infected with wild-type influenza, if treated, can develop *de novo* resistance and further spread the resistant pathogen. Our main purpose is to explore the impact of two important factors influencing treatment effectiveness: *i*) the relative transmissibility of the drug-resistant strain to wild-type, and *ii*) the frequency of *de novo* resistance. For the endemic scenario, we find a condition between these two parameters that indicates whether treatment regimes will be most beneficial at intermediate or more extreme values (*e.g.*, the fraction of infected that are treated). Moreover, we present analytical expressions for effective treatment regimes and provide evidence of its applicability across a range of modeling scenarios: endemic behavior with deterministic homogeneous mixing, and single-epidemic behavior with deterministic homogeneous mixing and stochastic heterogeneous mixing. Therefore, our results provide insights for the control of drug-resistance in influenza across time scales.

## Introduction

Rapid antigenic evolution in the influenza virus increases the likelihood of emergence of novel strains, against which little to no immunity may exist in the host population [1]–[4]. In this scenario, if vaccines are not yet available or non-pharmaceutical interventions have limited impact on disease containment, antiviral treatment plays a crucial role in the control of the disease [3], [5]–[7]. A critical constraint in the deployment of antivirals agents (*e.g.*, M2 inhibitors and neuraminidase inhibitors [8]) is the evolution of highly transmissible drug-resistant mutants [9]. Resistance decreases the effectiveness of chemotherapy in infected patients, prolonging recovery or leading to outright treatment failure [10]. Epidemics of untreatable strains have the potential to cause major morbidity and mortality [11]–[14], with significant economic costs for both the individual and for society writ large [15]. Consequently, public health policy has a growing need to understand the key factors that lead to the rise and spread of resistance, and to devise strategies that amplify the effectiveness of existing drugs, while halting the spread of resistance [16]–[19].

In addition to important precautionary measures, such as improvement of hospital counter-infection methods and regulation of antiviral use, mathematical models can be used to explore plausible competition scenarios between sensitive and resistant strains and the impact of treatment strategies on these dynamics [17], [19]–[22]. Previous models of the development of resistance of influenza to antiviral agents have focused on efforts to minimize the fraction of drug-resistant infections during an epidemic outbreak [5]–[7], [17] and to give recommendations that inform policy [8], [15], [18], [23]–[25]. However, the study of the long-term (endemic) dynamics of drug-resistance has received less attention [26].

The present work assesses the effectiveness of treatment at minimizing the total number of infections while halting the spread of drug-resistance, both from an endemic and a single-epidemic perspective. We focus our attention on two points: *i*) the relative transmissibility of the drug-resistant strain with respect to the wild-type (drug-sensitive) strain, and *ii*) the frequency of *de novo* resistance. Point *i*) is related to the fitness cost associated with the evolution of drug resistance, reflected in a reduced transmissibility of the drug-resistant pathogen relative to its wild-type counterpart [8], [27]. Recent evidence has demonstrated, however, that this reduction in fitness may be limited due to compensatory mutations which can restore fitness without loss of resistance-conferring genes [9], [28]. Point *ii*) represents the probability that treatment leads to resistance within the treated host (*de novo*). Both quantities are crucial in the population dynamics of drug-resistance, specially due to their variability within different epidemiological settings [8]. Nonetheless, their combined effect on the effectiveness of treatment regimes during influenza pandemics is not fully understood [5].

We build on a previous model [17] to examine these issues in the long-term (endemic disease prevalence) as well as in the short-term (single-epidemic). Lipsitch et al. [17] observed that intermediate levels of antiviral use are indicated to reduce the attack rate during an influenza pandemic. Complementing these results, we find that to effectively reduce the endemic levels of the wild-type and resistant strains, treatment regimes (*i.e.*, treated fraction) should be at intermediate levels if the resistant strain is highly transmissible and *de novo* resistance is rare. However, if resistance comes with a high fitness cost and *de novo* resistance is frequent, then higher levels of antiviral use may be preferable. In the single epidemic case we compare our optimal treatment regime with that of [17], showing that their relative effectiveness also depends on the strains’ relative transmissibility and the frequency of *de novo* resistance. Moreover, we demonstrate the applicability of our optimal treatment regimes by evidencing its effectiveness at quelling the spread of resistance when considering the effects of the stochasticity inherent to the transmission dynamics and the complex contact structure in the population.

## Methods

### Model Formulation

**Figure 1:**
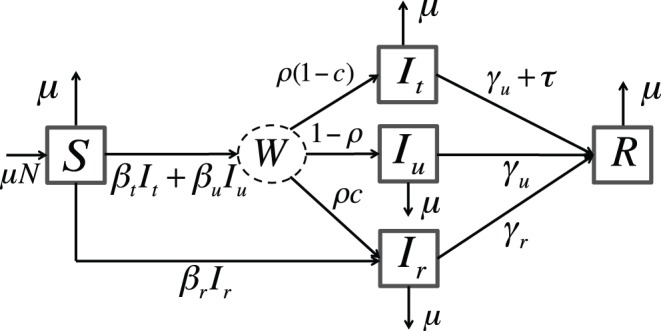
Compartmental Model for Eqs. (1)–(5).

We extend a version of the model in [17] to include demography (see Figure 1). Susceptible hosts, 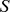, enter the population at a per-capita rate 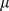 and die at rate equal to 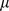, keeping the total population size, 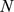, constant. Susceptible individuals can be infected by pathogens either sensitive or resistant to the available antiviral (this model does not include superinfection with both strains). A fraction 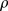 of patients infected with the wild-type strain are treated, and a fraction 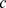 of those treated develop resistance *de novo*. Therefore, individuals infected with the wild-type strain are either untreated (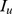), effectively treated (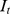), or resistant to treatment (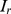). Infection with a resistant strain is either developed *de novo* or acquired from another resistant-infected individual. Susceptible individuals become infected at a rate proportional to the densities of susceptible and infected individuals, and to the transmission rates of each class, 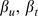, and 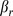, respectively. Untreated, treated, and resistant infected individuals recover at per-capita rates 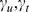, and 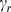, respectively. We assume no disease-induced mortality, and that the pathogen induces sterilizing immunity [21], [29].

The relative transmissibility of the resistant strain is defined as 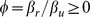. Successfully treated individuals: 1) are not more infectious: 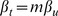, where 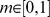 is the reduction in viral shedding [16], [30], [31], and 2) recover faster: 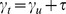, where 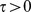 is the increase in recovery rate [8], [16], [32].

The ordinary differential equation (ODE) model describing these dynamics is
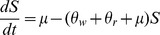
(1)

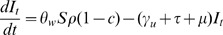
(2)

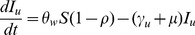
(3)

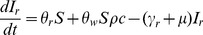
(4)

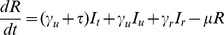
(5)with forces of infection 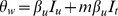 and 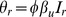. Note, we are modeling densities (*i.e.*, 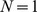). In what follows, let 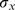 be the total per-capita rate out of class 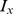, *i.e.*, 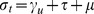, 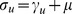, and 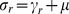.

#### Reproduction numbers

The basic reproduction number, 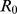, is the average number of secondary cases produced by a typical infected individual in a completely susceptible population. We find 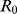 for each strain using the Next Generation Operator (NGO) method [33]. The non-zero eigenvalues of the NGO matrix
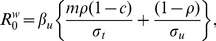
(6)

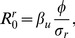
(7)are the reproduction number of the wild-type and resistant strains, respectively. Detailed derivations can be found in the Supporting Information (Text S1).

## Results

### Fixed Points and Bifurcation Analysis

The system (1)–(5) has three fixed points (FPs): 1) a disease free equilibrium (DFE); a FP where only the resistant strain persists (RFP); and a coexistence FP in which both strains coexist (CFP). Conceptually, these FPs represent: 1) eradication of both resistant and wild-type strain, eradication of the wild-type strain when treatment and/or relative transmissibility are high enough to allow persistence of the resistant strain; and coexistence of both strains due to low treatment and/or low fitness of the resistant strain, where typically the resistant strain persists at low levels. The FPs are:

**DFE:**

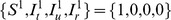
(8)


**RFP:**

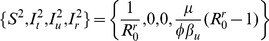
(9)


**CFP:**

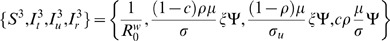
(10)where
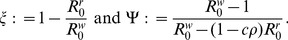
(11)The recovered class fraction in each case is given by 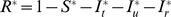. Comparing the susceptible steady states in (9) and (10) suggests that for the RFP, prevalent infections are attributable to the resistant strain, whereas for the CFP, the reproduction number of the wild-type strain determines how prevalent the disease is.

To be biologically significant (BS) the steady states have to lie in the set
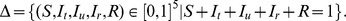



The RFP is BS if 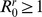. For the CFP, 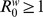 must hold so that 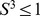. This also implies that the numerator of 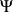 in (11) is positive. For 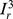 to be non-negative, the denominator of 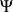 must be positive, *i.e.*, 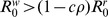, which implies 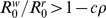. For 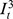 and 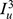 to be non-negative 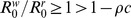 must hold. Therefore, the CFP is BS if
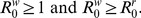
(12)


Thus, the two strains coexist if the wild-type strain is transmissible enough to be able to spread, and also more transmissible than the resistant strain.

#### Stability of fixed points

For the stability analysis of the FPs we study the eigenvalues of the matrix in the linearized system around the FPs: equilibria that have eigenvalues with negative real part are stable, whereas equilibria that have eigenvalues with positive real part are unstable [34]. We present here the results of the analysis; detailed analytic derivations can be found in the Text S1.

As expected, the DFE is globally stable if 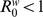 and 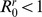. The RFP is locally stable if 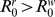. While determining the stability of the CFP is not analytically tractable, (12) states that the CFP is BS if 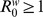 and 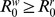. Thus, the conditions in (12) imply that neither the DFE nor the RFP are stable. We then conjecture that the CFP is BS and globally stable if (12) holds. Epidemiological arguments and numerical integrations support this hypothesis.

#### Bifurcation analysis

**Figure 2:**
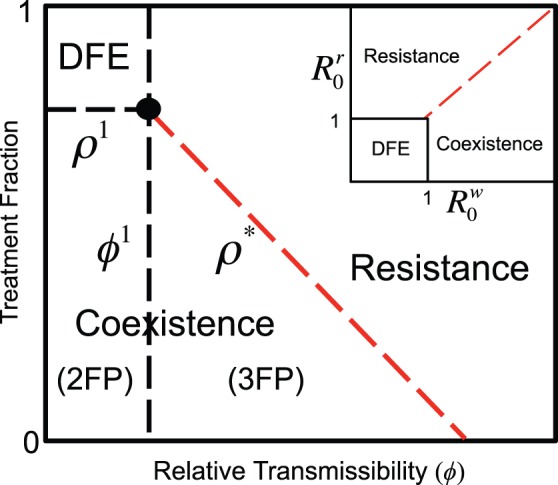
Stability regions in the (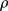, 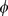) and (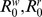) parameter space. Coexistence 2FP (CFP stable, DFE unstable); Coexistence 3FP (CFP stable, DFE unstable, RFP unstable); Resistance (DFE unstable, RFP stable). When the system crosses any of the region boundaries it experiences a transcritical bifurcation.

Depending on 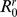 and 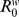, the system has one, two, or three BS FPs. Figure 2 features all four stability regions described above in the (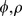) and the (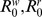) parameter space. The boundary of these regions can be found by solving 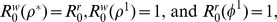 yielding
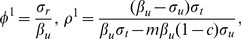
(13)and
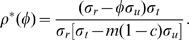
(14)


The boundaries are shown in Figure 2, where 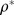 is the red-dashed line, 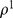 is the dashed and horizontal line, and 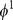 is the dashed and vertical line. The intersection of these curves (black dot) represents the overall disease threshold: any increase in 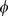 or decrease in 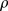 away from this intersection would result in an epidemic. Moreover, Eq. (13) shows that, for appropriate parameter values, increasing 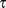 or decreasing 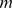, decreases the 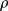-coordinate (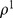) of the overall disease threshold point. Thus, increasing the recovery rate or decreasing the transmission rate of those treated, represents an epidemiological trade-off: it jointly expands the “DFE” and the “Resistance” stability regions, making it more likely for the system to either stay disease-free or give rise to prevalent resistance (see Figure S4, S5, S6, S7, S8 in Text S1 for details).

### Optimal Treatment Regimes

The main goal of this work is to derive treatment regimes (*i.e.*, treated fractions) that minimize the wild-type infections while restraining the spread of resistance. From the CFP in (10), it is clear that for very low treatment levels, the wild-type strain is prevalent in the population, and the resistant strain prevalence stays at minimal levels [10], *i.e.*, 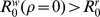. Additionally, treatment will reduce the viral shedding (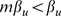) and increase the recovery rate by 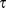, implying that 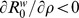. Thus, treating a larger proportion of the population will reduce the number of wild-type infected. However, it will also increase the number of *de novo* resistant cases, as well as the pool of susceptibles for the resistant strain to spread in.

These observations intuitively suggest that an effective treatment strategy should minimize 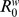 by increasing 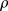, while keeping 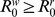. Formally,
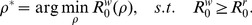
(15)


Since 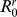 does not depend on 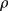 (Eq. (7)), and assuming 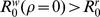, (15) can be solved by reducing 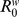 until 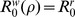. This equality yields 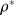 as in (14), a linearly decreasing function of the relative transmissibility, 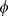 (see Figure 2).

However, (15) is inadequate since it does not consider the fitness advantage that development of *de novo* resistant cases give to the resistant strain. As 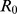 is the expected number of new cases produced by a typical infected person in a susceptible population, this quantity can be considered a measure of the fitness of a pathogen at the *population level*. Additionally, in our model, 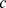 is directly related to the *within-host* fitness of the resistant pathogen. The overall fitness of the resistant strain is the added contributions of the fitness at the *population* and the *within-host* level. To estimate this overall fitness, assume, for the sake of clarity, 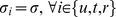. Let also 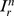 and 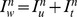 be the number of resistant and wild-type cases in the 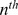 “epidemic generation” (with duration approximately 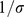) in a predominantly susceptible population. Defining 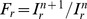 and 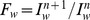 as the overall fitness of the resistant and wild-type strains, respectively, we obtain (see Text S1 for details):
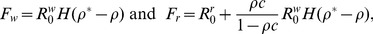
(16)where 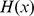 is the Heaviside step function (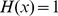 if 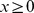, and 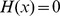 otherwise), *i.e.*, if 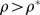, the wild-type strain goes extinct. It is then clear that 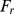 has an additional contribution from the *de novo* cases. More importantly, from the sole comparison of the reproduction numbers we cannot infer properly which strain will dominate, nor can we devise effective treatment regimes.

A more appropriate way to optimize the treatment regime is attained by focussing on the fixed points (FPs). The system has two FPs where the disease is endemic (RFP and CFP). On the one hand, if the CFP is stable, the optimal treatment regime, 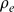, is *defined* as the fraction treated that yields the minimum number of wild-type infected, while the resistant is kept at lower endemic levels than the wild-type. Formally,
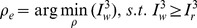
(17)where 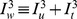. The regime 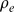 can then be found by solving for 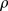 in 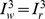 (see Eq. (19)).

On the other hand, if the RFP is stable, then treatment will have no effect on the prevalence of the resistant strain since 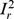 is not a function of 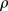. Two scenarios are then possible: **(A)** 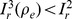, or **(B)** 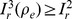. An assessment of these two scenarios yields our definition of overall *optimal* treatment regime 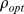: if **(A)** is true, 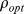 will minimize the endemic levels of the wild-type strain, while keeping the resistant strain at comparatively low levels; if **(B)** holds, 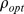 will transition the system to the RFP stability region. Hence, in case **(A)** it is best to maintain the system within the CFP limits, whereas in **(B)** the RFP will be preferred. The latter can be achieved by increasing 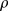 beyond 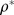. Formally,
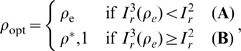



We now show that conditions **(A)** and **(B)** can be expressed in terms of our two key parameters: relative transmissibility, 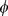, and the frequency of *de novo* resistance, 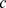. To find 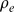 we solve for 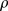 in 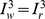, or,
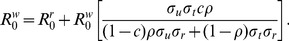
(18)


**Figure 3:**
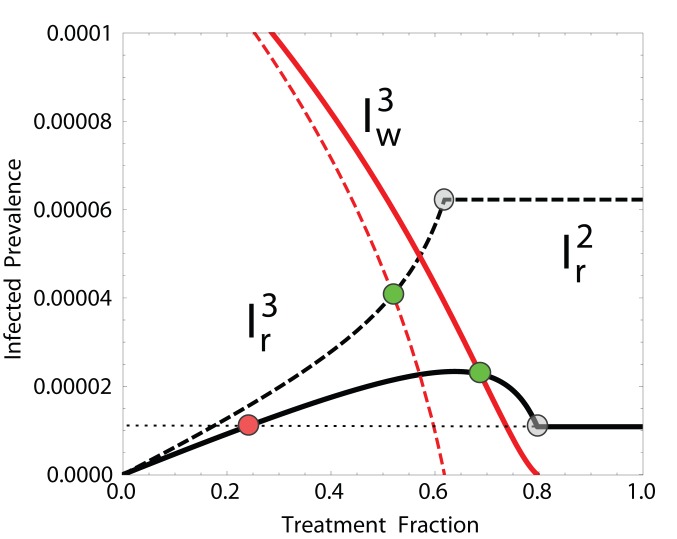
The two possible monotonicity behaviors of 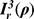. In black, 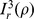 is concave for 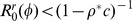 (solid lines) (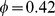), and monotonically increasing for 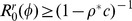 (dashed lines) (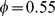). Red lines are the corresponding 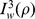 curves. The x-values of the green and gray dots represent 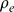 and 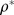, respectively, while the x-value of the red dot represents 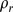. As 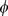 (or 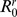) increases, the red dot moves rightward, surpassing the green dot (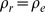), and eventually surpassing the gray dot as well (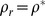). At this point, the system displays a transcritical bifurcation between the CFP and the RFP. Other parameters: 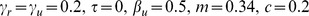. A large value of 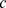 was used to magnify the difference between the two cases.

Within the CFP limits, (19) indicates when the overall fitness of both strains are equal (notice the similarity of the left hand side and the right hand side of (19) with, respectively, 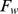 and 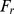 in (16), when 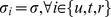). The explicit expression for 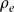 is given in the Text S1. Noteworthy, 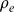 is the only value of 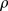 in 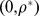 for which 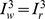. This claim is justified as follows: 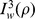 is a monotonically decreasing function of 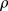 in 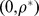, with 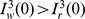 and 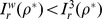. Additionally, 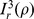 is either increasing or concave in 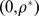 (see Figure 3). In both cases, 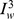 and 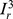 intersect at only one point (green dots in Figure 3). See Text S1 and Figure S10 for analytic details.

It is easy to show that 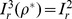 (gray dots in Figure 3). However, also 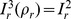, where
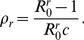
(19)


Then, if 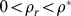, the term 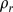 represents the treatment regime within the region of coexistence (CFP) for which the resistant strain is as prevalent as in the resistant-only stability region (RFP) (red dot in Figure 3). Additionally, it can be deduced from (20) that
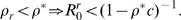
(20)


In the Text S1 we show that when (21) holds, 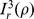 is concave for 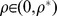. The concavity of 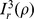 means, biologically, that the resistant strain prevalence is sustained largely by *de novo* resistant cases. Put differently, 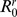 is not large enough for the resistant strain to self-sustain high levels of prevalence in the absence of treated wild-type infected.

Recalling that 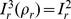 and 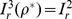, if 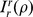 is concave for 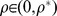 and 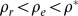, then 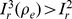 (condition **(B)**), indicating that the RFP is preferred over the CFP (solid curves in Figure 3). Furthermore, condition 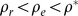 reduces to 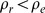 given that 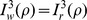 for 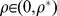. If instead 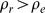, then condition **(A)** applies and keeping the system in the CFP while applying a treatment regime 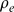 will be the best option (dashed curves in Figure 3). These observations along with expression (20) allow to restate conditions **(A)** and **(B)**, and therefore the optimal treatment, in terms of 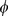 and 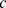 as
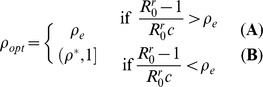



In case **(A)**, which corresponds to a high 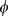 and low 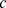 scenario, the CFP is stable with the wild-type and the resistant strains kept at low levels. In case **(B)** (*i.e.*, low 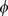 and high 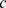), shifting the stability to the RFP is preferable since only the resistant strain will persist at low levels (see Figure 3). In other words, if the resistant strain features high relative transmissibility and resistance is rare, the best treatment regime would be at intermediate levels 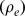; whereas if the opposite holds true, treating a larger fraction (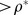) of the infected population is preferred.

**Figure 4:**
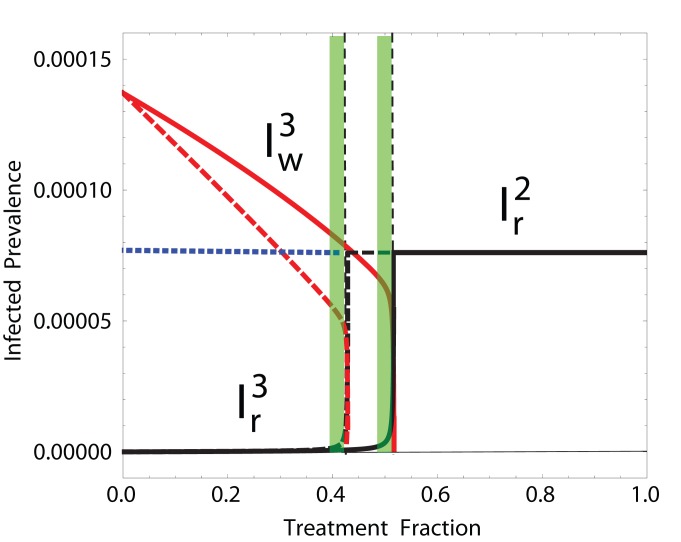
Effectiveness of 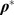 for 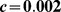 and 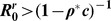. Prevalences 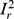 and 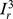 are depicted in black and 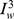 in red, for two different treatment recovery benefits (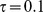, solid; 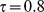, dashed). The RFP is unstable for 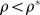 (blue dashed line). Strain dominance transition at 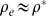 (vertical dashed lines). Optimal treatment regimes (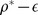) in green bands. Parameters: 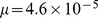, 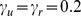, 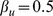, 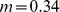, 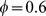 (see also Figure S9).

Recalling 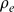 and 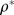 are found from (19) and 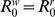, respectively, and noticing that as 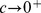 expression (19) reduces to 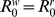, we conclude that 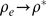 as 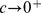. For this reason, when 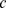 is small 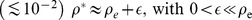, becomes a good treatment strategy if **(A)** holds. Moreover, as 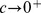, it is expected that **(A)** holds, at least in the epidemiologically interesting cases where 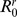 will likely be greater than 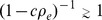 (*i.e.*, the resistant strain can emerge and spread in the population). In conclusion, when 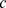 is small, then 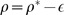 is a good treatment regime to minimize both the wild-type and the resistant strains (green bands in Figure 4).

Despite the large uncertainties in the frequency of patients that develop *de novo* resistance [8], 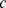 can be assumed to be relatively small. Figure 4 evidences how, for 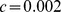 (as in [17]), 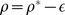 is the optimal treatment fraction: it diminishes the prevalence of the sensitive strain as much as possible, while hindering the emergence of the resistant strain. For low levels of treatment the CFP is stable: the wild-type strain prevails and the resistant strain (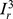) remains at low levels. As soon as 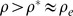, the resistant strain out-competes the wild-type strain. Expectedly, as treatment further reduces the infectious period (*i.e.* larger 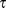, dashed lines), increasing treatment reduces the wild-type strain prevalence more effectively. In this case, the optimal levels of treatment are lower. A similar behavior is obtained when, instead of increasing 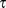, we reduce 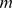 (reduction of viral shedding due to treatment).

#### Frequency of *de novo* resistance and endemic levels of resistance

**Figure 5:**
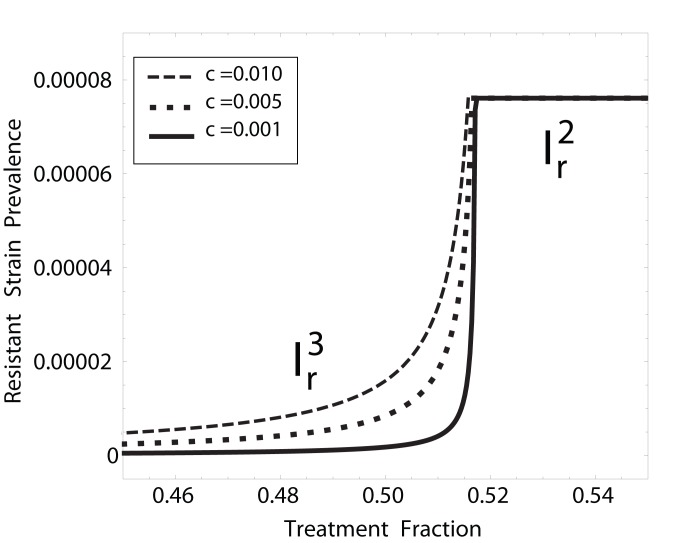
Resistant strain prevalence vs. treatment fraction. Smaller 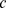 leads to more abrupt transitions from wild-type to resistant strains. Larger 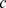 renders 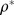 ineffective as a treatment regime.

We have shown how the frequency of *de novo* resistance, 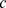, plays a crucial role in devising effective treatment strategies. In addition, we find that smaller values of 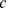 lead to more abrupt transitions from wild-type to resistant strains. In other words, the smaller the probability of developing *de novo* resistance, the faster the RFP gains stability when the system is close to the threshold 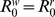 (Figure 5). Thus, for small 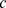, the system becomes more sensitive to variations in 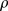, 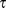, 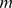, and 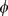 near this threshold. This represents a potentially dangerous scenario: if the likelihood of *de novo* resistance is small, a policy-maker might underestimate the prospects of resistance emergence and, consequently, increase treatment levels to eradicate the wild-type strain. However, if treatment is increased above 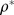, an abrupt transition may occur to a state where only resistant strains persist.

Mathematically, this “abrupt transition” can be justified as follows: if 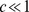, then 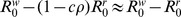; hence, when 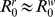, there is a “singularity” for 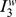 and 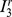 in the CFP (10). Biologically, it is clear from (16) that 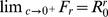 and 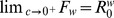. That is, as 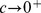, the reproduction numbers become the overall fitnesses of the strains, and 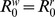 represents the condition for which both strains are equally fit. Thus, the resistant strain outcompetes the wild-type strain as 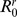 surpasses 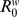.

### The Single Epidemic Case

Frequently, public health programs and interventions are designed to prevent the emergence of drug resistance within a single epidemic. To address this issue, we model a closed population (*i.e.*, 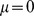 in model (1)–(5)), and examine again the role of *i*) the relative transmissibility (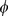) and *ii*) the frequency of *de novo* resistance (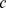) on the effectiveness of treatment regimes. In this assessment we focus on the final epidemic size (FS), defined as the proportion of the population infected during the epidemic. As in [17], we introduce the following correction to our numerical integrations: if 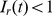 then 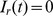. This prevents spurious results induced by the transmission of “non-cases” (since 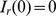, initially 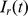 can only increase due to *de novo* resistant cases; given that 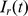 is continuous in the ODE framework, the condition above avoids that a fraction of a *de novo* resistant case can cause a direct resistant infection). Throughout this section the following parameters are fixed: 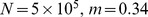, 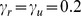, 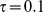, 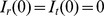 and 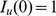.

**Figure 6:**
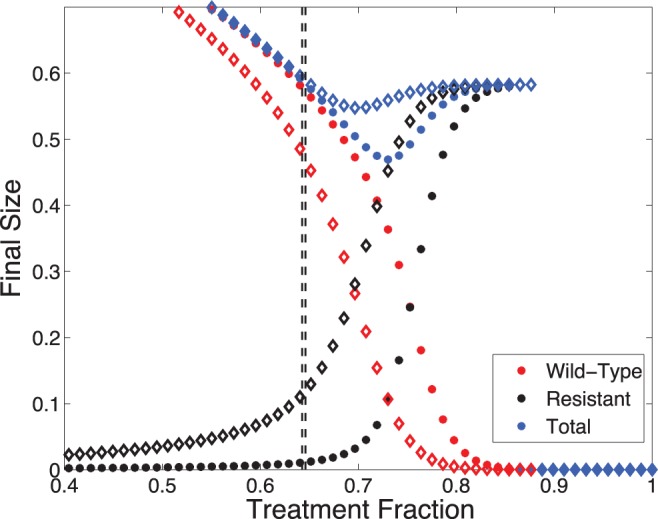
Final epidemic size of both strains vs. 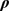. (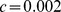 as asterisks and 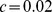 as diamonds) Vertical dashed lines represent 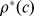; higher 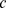, lower 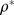. Note that 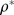 is effective in halting the spread of resistance but not in reducing the total FS. Also, for larger 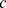, 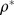 loses its effectiveness in avoiding the spread of resistance. Other parameters: 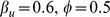.

Figure 6 shows a feature demonstrated previously [6], [17], [22]: the existence of an “optimal” level of treatment for which the total FS is minimized. We can readily see this minimum is a function of 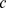: as 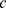 increases, the dip in the combined FS curve vanishes. Furthermore, the treatment regimes that minimize the total FS, are not optimal in terms of avoiding the emergence of resistance. Let 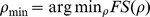. We find that 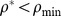, where as before 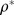 satisfies 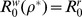. That is, the minimum in the FS is reached when resistance has already significantly spread in the population. Additionally, Figure 6 shows that for larger 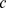 (diamond curves), 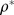 represents a value of the treatment fraction for which the resistant strain has already spread considerably throughout the population. This suggests that, as in the endemic case, the effectiveness of 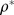 depends on the frequency of *de novo* resistance: as 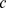 increases, the validity of 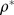 becomes compromised (notice similarity in black curves of Figures 5 and 6).

Notice also in Figure 6 that the epidemic is eradicated if 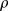 exceeds 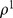 (Eq. (13)), where 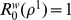. That is, when the treatment fraction is large enough to rapidly halt the spread of the wild-type strain, the resistant strain will not emerge. This is possible, in part, given our assumption that treatment is implemented early in the epidemic (*i.e.*, 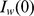 is small). In conclusion, if 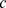 is relatively small and treatment is put in place later in the epidemic or it cannot surpass 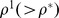, then 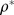 will ensure minimal spread of the resistant strain.

**Figure 7:**
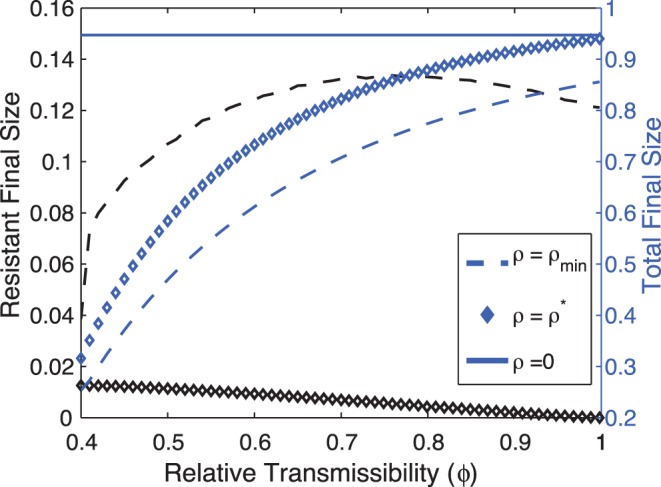
Final Sizes vs. relative transmissibility for 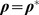 and 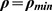. The figure shows the effectiveness of 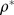 and 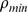 vs. the relative transmissibility. For any value of 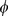, 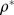 is more effective than 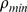 at avoiding the spread of resistance in the population (black diamond vs. black dashed curves). However, 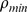 is more efficacious at reducing the overall epidemic (blue dashed vs. blue diamond). Solid line corresponds to 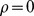. Other parameters: 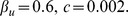

We now wish to contrast the effectiveness of 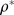 and 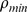 as a function of the relative transmissibility 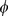, assuming relatively low frequency of *de novo* resistance 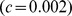. Figure 7 shows the FSs (due to resistant strain (black) and total (blue)) vs. 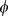 for 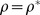 and 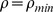. A treatment regime 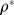 would “prioritize” the avoidance of resistance, while compromising the reduction of the overall epidemic; conversely, 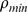 will, by definition, “prioritize” the minimization of the total epidemic size, while disregarding the spread of resistance. As a result, 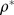 is more effective than 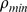 at halting the spread of resistance in the population, whereas 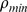 is a better option to reduce the overall epidemic. Moreover, since 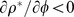 (see (14)), as 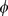 increases, a treatment regime 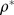 will systematically diminish the spread of resistance by reducing the treated fraction. Consequently, for higher 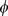, 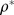 will have minimal effects on reducing the total epidemic size (compare the diamond with the horizontal blue line, where no treatment is applied). Therefore, the decision to use 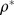 or 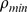 as a treatment regime will mainly depend on how policy makers balance a larger epidemic produced largely by the wild-type strain, with minimal resistant cases (using 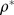, for which we have a better biological and mathematical understanding), versus a smaller overall epidemic with higher resistance incidence (using 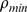).

To summarize, when the fraction of *de novo* resistant cases and the relative transmissibility are rather small, 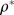 constitutes a useful quantity for treatment policies in a single epidemic outbreak provided it can contain the overall epidemic while restraining the spread of resistance in the population.

#### Relative transmissibility and non-pharmaceutical interventions

It is likely that treatment alone cannot completely quell an emerging epidemic [35]. In such cases, non-pharmaceutical interventions (*e.g.*, social distancing, case isolation, travel restrictions) could help to significantly mitigate the extent of the epidemic [3], [8], [16]. These can affect the transmissibility of the wild-type and the resistant strain while maintaining the relative transmissibility of the latter (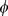). Here we investigate the competition dynamics between the wild-type and the resistant strain as a function of 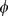, and the transmissibility of the wild-type strain 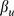 (which varies due to non-pharmaceutical interventions) under different treatment regimes.

**Figure 8:**
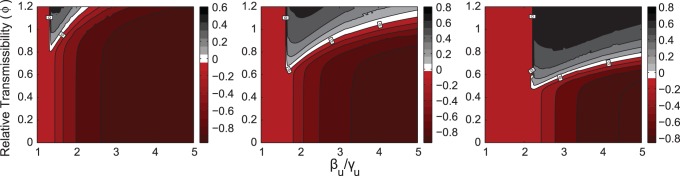
FS_
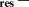
_FS_
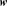
_ in the (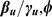) parameter space. 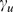
 is fixed, and 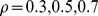 (left to right). Gray-black regions are dominated by the resistant strain. As treatment increases the resistant strain 1) benefits from higher wild-strain transmissibility, 2) increases the range of relative transmissibility for which it can spread, and 3) expands the region in which it can extensively spread (black region).

The total FS is comprised by the resistant-strain cases (FS_
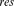
_) plus the wild-type cases (FS_
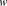
_). To determine the dominant strain, we compare FS_
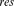
_ and FS_
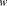
_. Figure 8 shows numerical results of FS
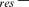
FS_
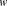
_ in the (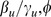) parameter space (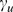 fixed). For instance, if FS_
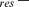
_FS_
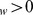
_ (gray-black region), the resistant strain is accountable for more cases than the wild-type strain. The wild-type dominated region is in red.

The resistant strain can only spread in the 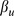 region for which the wild-type strain significantly spreads: notice in each graph, a vertical light-red region where only the wild-type strain minimally spreads, and to its right we see regions of coexistence. The value of 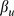 defining the split of these two regions, 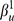, is such that 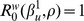; if 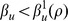, the wild-type strain will only generate few infections and consequently the resistant strain will mainly be in rare *de novo* resistant cases. A similar consideration was presented in Figure 6.

In general, for lower 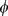 the resistant strain cannot spread, while the wild-type strain produces an increasingly larger number of infections as 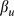 increases. As treatment (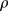) increases, the resistant-dominated region shifts to higher values of 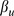, while expanding the range of 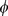 for which it can significantly spread (darker areas).

These observations suggest that if the wild-type strain features relatively low transmission, the best strategy to contain both strains is to treat “hard and early”. However, if the transmissibility is higher and the fitness cost of resistance is low, then this strategy can have devastating consequences as the resistant strain can infect a large fraction of the population. This demonstrates the importance of effective non-pharmaceutical interventions that could reduce 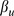.

For larger values of 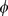 an interesting process occurs. Starting from low 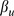 the wild-type dominates. As 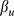 increases – crossing the “vertical” null isocline where both FSs are equal – the resistant strain begins to prevail, until crossing the “slant” null isocline where the wild-type strain starts to regain its dominance. A possible explanation for this dominance shift is that as the wild-type strain becomes more transmissible, it depletes the pool of susceptibles too quickly, leaving the resistant strain with few individuals to infect once it emerges. However, for even larger 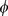 and high 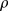, increasing 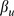 also increases the FS of the resistant strain. In this scenario, the relative transmissibility is so high that even if the wild-type strain can spread rapidly, the resistant strain will eventually “catch up” and outcompete it.

Thus, when trying to predict the outcome of the competition dynamics between wild-type and resistant strains, knowing the relative transmissibility of the latter is not sufficient. One must also know the actual value of its transmissibility. In the endemic case, however, when treatment is fixed, the relative transmissibility completely determined which strain ultimately dominated (Figure 2). These considerations complement observations made in [17].

### The Impact of Contact Structure

Two strong simplifications made in our model were to ignore the complex contact structure of human populations and the stochastic nature of the transmission and *de novo* resistance dynamics. While these assumptions allowed us to obtain closed-form solutions for effective treatment regimes, the social network underlying the epidemic process is known to have non-trivial effects on transmission dynamics [21], [22], [36], [37]. In this section we use a model equivalent to (1)–(5) that features contact structure [38] and stochasticity [39]. We again assume 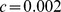, and utilize Monte-Carlo (MC) simulations to assess the effectiveness of 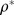 (Eq. (14)) in single epidemic situations.

**Figure 9:**
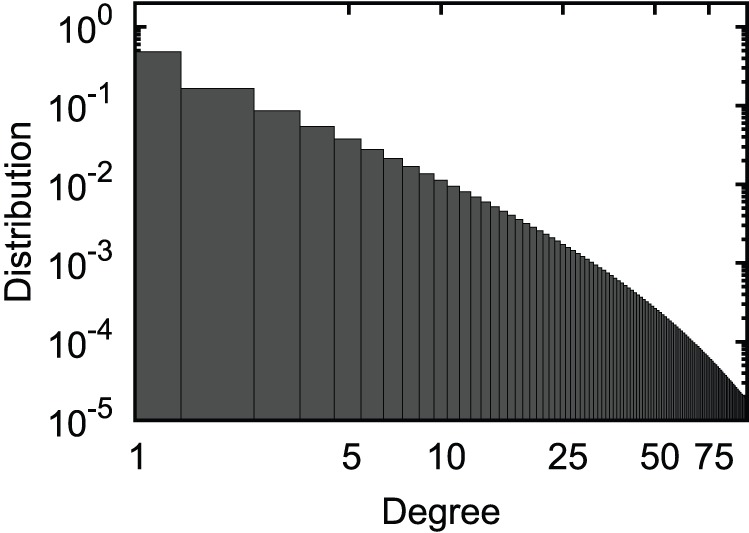
The fat-tailed degree distribution (contact per individual) with power-law tail and exponential cut-off. Used to generate heterogeneous networks for the MC simulations.

To perform MC simulations of the model, we have generated networks of size 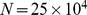 with fat-tailed degree distributions 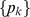 (distribution of number of contacts per individual, shown in Figure 9), via the Configuration Model algorithm [40]. For every generated network, a randomly chosen individual is infected with the wild-type strain and the dynamics are then simulated in discrete time:

- each time step, every susceptible neighbor 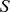 of every infectious individual 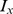 is infected with probability 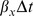;
- wild-type infections are treated with probability 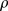, leading to resistance-conferring mutation with probability 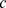;
- each time step every infectious individual 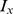 recovers with probability 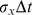 (with 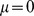).

**Figure 10:**
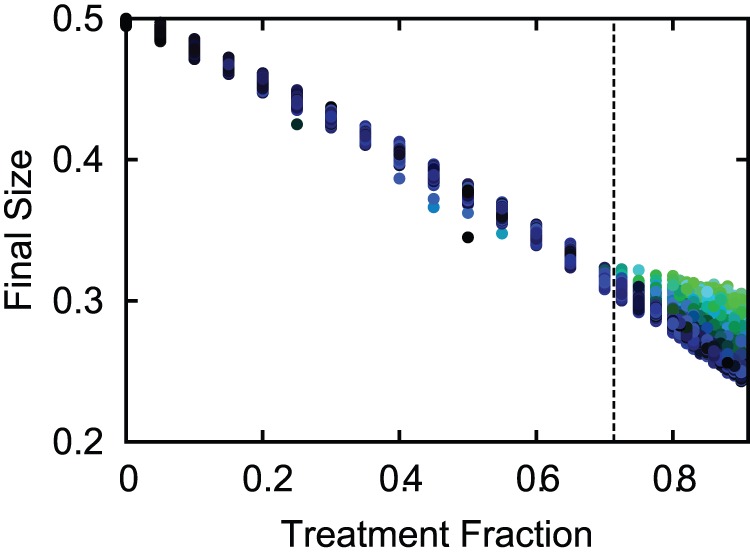
Monte Carlo simulations on a network with heterogeneous contact structure. (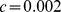). Every point represents one of over 10,000 simulations on networks of size 250 000, with color indicating the proportion of resistance in the FS (from black, 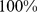 wild-type, to green, 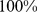 resistant). 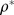 (Eq. (14)) is shown in dashed black line. The effectiveness of 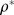 is robust to stochasticity and heterogeneous contact structures. Other parameters: 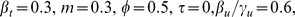 and 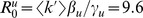, where 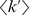 is the average excess degree in the network [39].

Figure 10 shows the variation in the final epidemic size (FS) of the system due to the contact heterogeneity and the inherent stochasticity of the disease and pathogen mutation processes. The worst-case scenarios (the highest FS obtained for a given value of 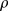) qualitatively follow the same behavior as the ODE model above (blue curves in Figure 6). More importantly, the predicted optimal treatment fraction 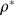 provides a good approximation to what could be considered the best treatment plan, yielding the lowest total FS while halting the spreading of resistance (Figure 10, greener dots). As in the deterministic case, for 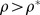, resistance spreads widely. Hence, when the frequency of *de novo* cases is small, the efficacy of the treatment fraction 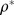 to minimize both the epidemic size and the risks of resistance emergence, is robust to both the heterogeneity of population structure as well as the stochasticity of transmission and mutation dynamics.

## Discussion

The rapid development of an effective vaccine against an emerging novel influenza virus presents considerable challenges. Thus, antiviral agents could play a central role as a first-line defense against emerging epidemics of influenza. The large-scale use of these drugs could, in turn, select for the evolution of drug-resistant strains [8], making the strategic distribution of antivirals essential in quelling the spread of drug-resistance while limiting the overall epidemic size. In this work we have discussed the influence of two key parameters on the effectiveness of treatment: the relative transmissibility of the drug-resistant strain (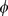), and the frequency of *de novo* resistance (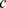). We extended a previous model [17] to include demography and performed analytical calculations of the reproductive numbers, stability of the fixed points, and conditions for the exclusion or coexistence of resistant and wild-type pathogen strains.

In the endemic case we found that, depending on the values of 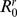 (or equivalently 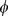) and 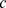, the optimal treatment regime will be at intermediate (case **(A)** for high 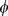 and low 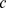 ) or more extreme values (case **(B)** for low 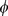 and high 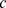). Intuitively it is clear that if the resistant strain is highly transmissible (high 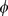), then treatment should be moderate in order to limit the selective advantage of drug-resistant phenotypes. Conversely, if the resistant strain is weakly transmitted (low 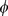), then more intense treatment regimes are preferred since resistance-only endemic levels will be relatively low. These recommendations are valid as long as infections with a wild-type or a resistant strain represent the same harm to the host (*e.g.*, strains with similar infectious periods and virulence). In addition, we also remarked that when 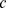 is low, the optimal treatment regime can be approximated by 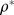. In the single epidemic case, numerical simulations also suggest that if 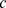 and 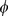 are low, 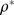 is still a useful quantity when designing treatment strategies. However, in contrast to the endemic case, knowing the relative transmissibility of the resistant strain is not enough to predict the final outcome of the competition between the two strains. In this case, the strain that successfully spreads first has a significant impact on which strain infects more individuals during the epidemic. Our results also indicate that early and high treatment regimes are most effective at reducing the number of infections while hindering the rise of resistance, when the transmissibility of the wild-type strain is relatively low. This emphasizes the importance of non-pharmaceutical interventions aimed at reducing the transmission rate of the disease.

Further, we showed that for small 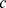, the parameter 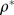 is robust to the presence of contact heterogeneity and stochasticity, as it still minimizes both the epidemic size and the risks of resistance emergence. This reinforces the public health implications of the effective treatment expressions derived herein.

An interesting similarity across time scales is the impact of the frequency of *de novo* resistance on 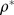: as 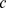 increases, the effectiveness of 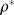 becomes compromised. While we give mathematical and biological arguments for this property, the inherent uncertainty in the empirical values of 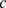 make this observation potentially relevant to the designing of treatment strategies [8].

Our model, like any other, is not exempt of simplifying assumptions, or uncertainties about the model parameter values and transmission dynamics of wild-type and resistant strains. Thus, rather than providing specific quantitative recommendations for treatment policies, we emphasize the qualitative character of our observations. Moreover, we recognize that even if these uncertainties were resolved, we still face ethical issues when deciding to implement treatment policies based on our recommendations; *e.g.*, treat *only* a certain fraction of those infected if relative transmissibility is high and *de novo* resistance is unlikely. This is a difficult case for the public health planner, and the choice is left to them. If relative transmissibility is low and *de novo* resistance is more likely, then our recommendations are less controversial: treat people as they come in based on their clinical profile. In terms of the assumptions made in our analysis, we considered that treatment and *de novo* resistance happen immediately after infection. In the Text S1 we present a model that features stage progressions (treatment and *de novo* resistance occur at certain rates rather than instantaneously) and show that its dynamics are analogous to those presented here (see Figure S1, S2, S3). We also assumed that the fraction of treated individuals can, with no regard to economic and social costs, attain any value between 0 and 1, and remain constant throughout time. This is generally not true as treatment availability and costs vary with time and socioeconomic context (models in [5], [6], [41], [42] explore different time-dependent treatment regimes). We have considered a model with equal birth and death rates, thus, it may also be important to study the impact of demographics on the effectiveness of treatment regimes, though less so in the single epidemic case. We have also excluded coinfection with both strains, which is known to affect the evolution of the influenza virus (*e.g.*, viral reassortment [4]), and could in turn influence the development of drug-resistant phenotypes. We suspect that accounting for coinfection might lead to new and interesting dynamics.

Our results shed light on the epidemiological impact of the interplay between treatment regimes and relative transmissibility of a strain of influenza resistant to antiviral treatment and the frequency of *de novo* resistance, both aspects which are difficult to assess empirically. These findings could have important implications for the strategic distribution of antivirals in a population in response to the emergence of a novel influenza strain.

## Supporting Information

Figure S1 **Compartmental diagram for the analogous model.**
(EPS)

Figure S2 **Comparison of the two models in the endemic case.**
(EPS)

Figure S3 **Comparison of the two models in the single epidemic case.**
(EPS)

Figure S4 **Transcritical Bifurcation between DFE and CFP.**
(EPS)

Figure S5 **Transcritical Bifurcation between RFP and CFP.**
(EPS)

Figure S6 **Transcritical Bifurcation between the DFE and the RFP.**
(EPS)

Figure S7 **Transcritical Bifurcation between RFP and CFP.**
(EPS)

Figure S8 **Stability behavior of the system.**
(EPS)

Figure S9
**Prevalence as a function of 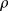 and 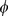.**
(EPS)

Figure S10

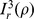
 **for 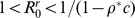 and for 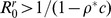.**
(EPS)

Text S1 **Analytical derivation of reproduction numbers; analogous model; analytical derivations regarding the stability of the system; and analytical derivations regarding the optimal treatment regimes.**
(PDF)
