# Supplementary material for: Optimizing Treatment Regimes to Hinder Antiviral Resistance in Influenza across Time Scales
Source: PLoS One. 2013 Mar 29;8(3):e59529. doi: 10.1371/journal.pone.0059529 (PMC3612110; doi:10.1371/journal.pone.0059529)
Supplement: Text S1 — Analytical derivation of reproduction numbers; analogous model; analytical derivations regarding the stability of the system; and analytical derivations regarding the optimal treatment regimes. (PDF) [file pone.0059529.s011.pdf]

Optimizing Treatment Regimes to Hinder Antiviral Resistance in Influenza Across Time Scales

Oscar Patterson-Lomba[1*], Benjamin M. Althouse[2], Georg M. Goerg[3], Laurent Hébert-Dufresne[4]

1 Mathematical, Computational, and Modeling Sciences Center, School of Human Evolution and Social Change, Arizona State University, Tempe, AZ, USA

2 Department of Epidemiology, Johns Hopkins Bloomberg School of Public Health, Baltimore, MD, USA

3 Department of Statistics, Carnegie Mellon University, Pittsburgh, PA, USA

4 Département de Physique, de Génie Physique, et d'Optique, Université Laval, Québec (Québec), Canada

∗ **E-mail: opatters@asu.edu**

1 R_0 and the Next Generation Operator

The basic reproductive number, R_0, is the average number of secondary cases produced by a typical infected individual in a completely susceptible population. We proceed to compute R_0 using the Next Generation Operator based on the approach in [1]. Considering only the infective states $\{I_t, I_u, I_r\}$, we obtain the reduced system

$$\frac{dI_i}{dt} = F_i - V_i, \quad i \in \{t, u, r\} \tag{1}$$

where

$$F = \begin{pmatrix} S(I_t m\beta_u + I_u\beta_u)\rho(1-c) \\ S(I_t m\beta_u + I_u\beta_u)(1-\rho) \\ SI_r f\beta_u + S(I_t m\beta_u + I_u\beta_u)c\rho \end{pmatrix} \quad \text{and} \quad V = \begin{pmatrix} I_t(\tau + \gamma_u + \mu) \\ I_u(\gamma_u + \mu) \\ I_r(\gamma_r + \mu) \end{pmatrix} \tag{2}$$

The Jacobian matrices of both F and V, evaluated at the disease free equilibrium (DFE)

$$X_0 = (S^* = 1, I_t^* = 0, I_u^* = 0, I_r^* = 0), \tag{3}$$

are

$$DF(X_0) = \begin{pmatrix} m\beta_u\rho(1-c) & \beta_u\rho(1-c) & 0 \\ m\beta_u(1-\rho) & \beta_u(1-\rho) & 0 \\ m\beta_u c\rho & \beta_u c\rho & f\beta_u \end{pmatrix} \tag{4}$$

and

$$DV(X_0) = \begin{pmatrix} \tau+\gamma_u+\mu & 0 & 0 \\ 0 & \gamma_u+\mu & 0 \\ 0 & 0 & \gamma_r+\mu \end{pmatrix} \tag{5}$$

The Next Generator Operator (NGO) matrix is defined as $M = DF \times DV^{-1}$. Using the inverse

$$DV^{-1} = \begin{pmatrix} \frac{1}{\tau+\gamma_u+\mu} & 0 & 0 \\ 0 & \frac{1}{\gamma_u+\mu} & 0 \\ 0 & 0 & \frac{1}{\gamma_r+\mu} \end{pmatrix} \tag{6}$$

we obtain

$$M = \begin{pmatrix} \frac{m\beta_u\rho(1-c)}{(\tau+\gamma_u+\mu)} & \frac{\beta_u\rho(1-c)}{(\gamma_u+\mu)} & 0 \\ \frac{m\beta_u(1-\rho)}{(\tau+\gamma_u+\mu)} & \frac{\beta_u(1-\rho)}{(\gamma_u+\mu)}, & 0 \\ \frac{m\beta_u c\rho}{(\tau+\gamma_u+\mu)} & \frac{\beta_u c\rho)}{(\gamma_u+\mu)} & \frac{f\beta_u}{\gamma_r+\mu} \end{pmatrix} \tag{7}$$

The eigenvalues of M are

$$\lambda_1 = R_0^w = \beta_u \left\{ \frac{m\rho(1-c)}{\gamma_u+\tau+\mu} + \frac{(1-\rho)}{\gamma_u+\mu} \right\}, \tag{8}$$

$$\lambda_2 = R_0^r = \beta_u \frac{\phi}{\gamma_r+\mu}, \tag{9}$$

$$\lambda_3 = 0. \tag{10}$$

where R_0^w and R_0^r are the reproductive number of the wild-type and resistant strains, respectively. The condition $R_0^w = R_0^r$ yields

$$\rho^* = \frac{[(\gamma_r+\mu)-\phi(\gamma_u+\mu)](\gamma_u+\mu+\tau)}{(\gamma_r+\mu)[(\gamma_u+\mu+\tau)-m(1-c)(\gamma_u+\mu)]} \tag{11}$$

2 Analogous Model

In the original model it is assumed that treatment and *de novo* resistance happen immediately after infection. Here we present a model that features stage progressions (treatment and *de novo* resistance occur at certain rates rather than instantaneously). Susceptible hosts, S, enter the population at a per-capita rate μ. The per-capita death rate of all classes is also μ. Since the population is kept constant, we assume $N = 1$. Susceptible individuals can be infected by either a wild-type or drug-resistant strains, progressing into the I_u and I_r classes, respectively. Those infected with the wild-type strain recover at rate γ_u, or get treated at rate α, entering the treated I_t class. From this class, individuals recover at rate $\gamma_u + \tau$, or develop *de novo* resistance at rate ν. Those infected with the resistant strain recover at rate γ_r. The pathogen induces sterilizing immunity.

The ordinary differential equation (ODE) model describing the above dynamics is (see Figure S1)

$$\begin{aligned}
\frac{dS}{dt} &= \mu - (\theta_w + \theta_r + \mu)S \\
\frac{dI_u}{dt} &= \theta_w S - (\gamma_u + \mu + \alpha)I_u \\
\frac{dI_t}{dt} &= \alpha I_u - (\gamma_u + \tau + \mu + \nu)I_t \\
\frac{dI_r}{dt} &= \theta_r S + \nu I_t - (\gamma_r + \mu)I_r \\
\frac{dR}{dt} &= (\gamma_u + \tau)I_t + \gamma_u I_u + \gamma_r I_r - \mu R
\end{aligned}$$

with forces of infection $\theta_w = \beta_u I_u + m\beta_u I_t$ and $\theta_r = \phi\beta_u I_r$, where $\phi = \beta_r/\beta_u$ and $m = \beta_t/\beta_u$.

Therefore, in this model $1/\alpha$ represents the average amount of time a wild-type infected, that will be treated, spends untreated, while $1/\nu$ represents the average amount of time it takes for those treated that will develop *de novo* resistance to actually become resistant to treatment. Moreover, the fraction of wild-type infections treated, ρ, and the fraction of those treated that develop *de novo* resistance, c, are

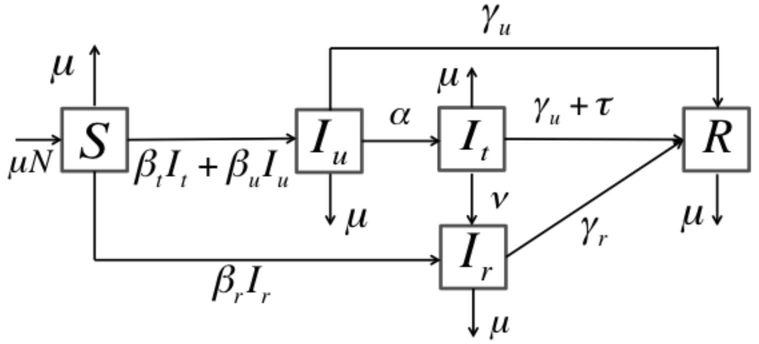

Figure S1. Compartmental diagram for the analogous model.

given by

$$\rho = \frac{\alpha}{\alpha + \gamma_u + \mu} \quad \text{and} \quad c = \frac{\nu}{\nu + \gamma_u + \tau + \mu}.$$

Notice that we have intentionally used the same terminology as in the manuscript.

In this model, the basic reproduction numbers are given by

$$\begin{aligned} R_0^w &= \beta_u \left\{ \frac{1}{\alpha + \gamma_u + \mu} + m \left(\frac{\alpha}{\alpha + \gamma_u + \mu} \right) \left(\frac{1}{\nu + \gamma_u + \tau + \mu} \right) \right\}, \\ R_0^r &= \beta_u \frac{\phi}{\gamma_r + \mu} \end{aligned} \tag{12}$$

where R_0^w and R_0^r are the reproductive number of the wild-type and resistant strains, respectively. Note that $1 - \rho = (\gamma_u + \mu)/(\alpha + \gamma_u + \mu)$, and $1 - c = (\gamma_u + \tau + \mu)/(\nu + \gamma_u + \tau + \mu)$. Thus, we can rewrite the reproduction numbers in (12) as

$$\begin{aligned} R_0^w &= \beta_u \left\{ \frac{1 - \rho}{\gamma_u + \mu} + m\rho \frac{1 - c}{\gamma_u + \tau + \mu} \right\}, \\ R_0^r &= \beta_u \frac{\phi}{\gamma_r + \mu} \end{aligned}$$

which coincide with the reproduction numbers presented in the manuscript, i.e., expressions (8) and (9). To compare the temporal dynamics and final states of the original model (the one in the main text) and the model with stage progression, Figure S2 and Figure S3 show numerical integrations with the similar parameters (we have used a higher birth/death rate to increase the endemic equilibria and better

convey our message) as in the original model for the endemic and single epidemic case, respectively. The qualitative behavior is quite similar. Note that the endemic equilibria and the total final sizes are the same in both models, which is expected since the two models have equal reproduction numbers. Thus, the two models are qualitatively analogous.

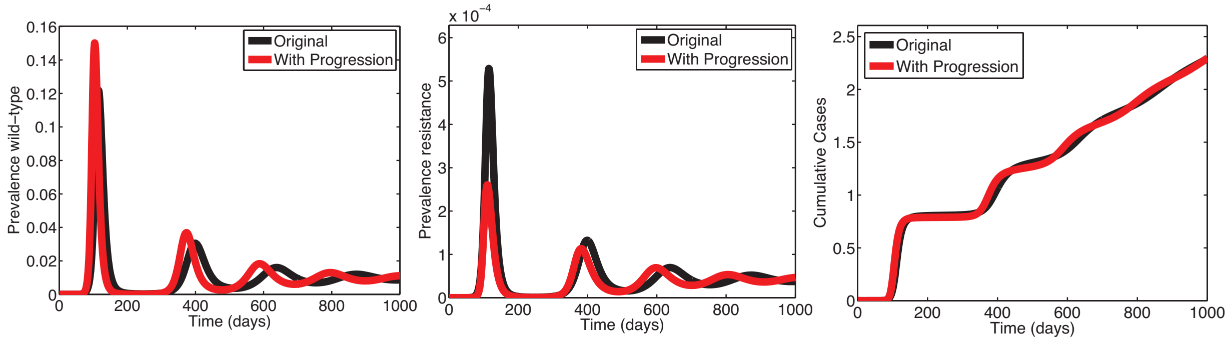

Figure S2. Comparison of the two models in the endemic case. Although their temporal dynamics are not equivalent, their endemic equilibria coincide.

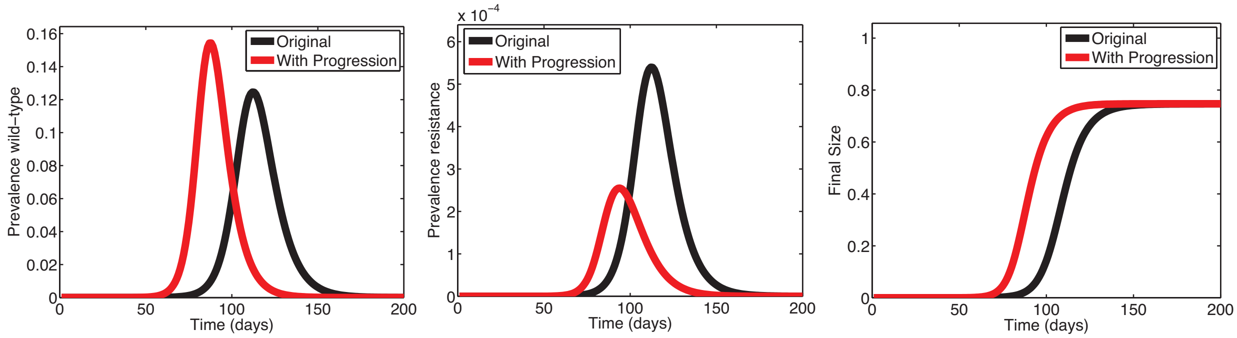

Figure S3. Comparison of the two models in the single epidemic case. The original model over estimates the resistance prevalence in comparison with the model with stage progression. The total final sizes coincide.

3 Stability of Fixed Points

Linearizing the system around the steady states, we found the respective eigenvalues λ_i, $i = 1, 2, 3, 4$. The sign of their real part determines the stability of the steady states [2].

3.1 DFE

For the DFE

$$\lambda_1^1 = -\mu \tag{13}$$

$$\lambda_2^1 = (\gamma_r + \mu)(R_0^r - 1) \tag{14}$$

$$\lambda_3^1 = -(\gamma_u + \mu) - \frac{1}{2}[\tau - (1-\rho)\beta_u - (1-c)\rho\beta_t] - \sqrt{\frac{1}{4}[\tau - (1-c)\rho\beta_t - (1-\rho)\beta_u]^2 + (1-\rho)\tau\beta_u} \tag{15}$$

$$\lambda_4^1 = -(\gamma_u + \mu) - \frac{1}{2}[\tau - (1-\rho)\beta_u - (1-c)\rho\beta_t] + \sqrt{\frac{1}{4}[\tau - (1-c)\rho\beta_t - (1-\rho)\beta_u]^2 + (1-\rho)\tau\beta_u} \tag{16}$$

The first eigenvalue is always negative; $\lambda_2^1 < 0$ if $R_0^r < 1$.

λ_3^1 and λ_4^1 have zero imaginary part if the term inside the square root is positive. The stability of the DFE depends on the respective signs. We prove below that $\lambda_3^1 < 0$ and $\lambda_4^1 < 0$, if $R_0^w < 1$. Thus, as expected, the DFE is stable if $R_0^w < 1$ and $R_0^r < 1$.

The stability of the DFE depends on their respective signs. Lets momentarily define

$$A := \frac{1}{2}[\tau - (1-\rho)\beta_u - (1-c)\rho\beta_t] \tag{17}$$

Then

$$\lambda_3^1 = -(\gamma_u + \mu) - A - \sqrt{A^2 + (1-\rho)\tau\beta_u} \tag{18}$$

$$\lambda_4^1 = -(\gamma_u + \mu) - A + \sqrt{A^2 + (1-\rho)\tau\beta_u} \tag{19}$$

Therefore, $\lambda_3^1 < 0$ and $\lambda_4^1 < 0$ simultaneously if

$$\begin{aligned}
(\gamma_u + \mu) + A &> \sqrt{A^2 + (1-\rho)\tau\beta_u} \\
\implies [(\gamma_u + \mu) + A]^2 &> A^2 + (1-\rho)\tau\beta_u \\
\implies (\gamma_u + \mu)^2 + 2A(\gamma_u + \mu) &> (1-\rho)\tau\beta_u \\
\implies (\gamma_u + \mu) + 2A &> \tau\frac{(1-\rho)\beta_u}{\gamma_u + \mu}
\end{aligned}$$

Substituting A and defining $R_0^u = \dfrac{(1-\rho)\beta_u}{\gamma_u+\mu}$ and $R_0^t = \dfrac{\rho(1-c)\beta_t}{\gamma_u+\tau+\mu}$, with $R_0^w = R_0^t + R_0^u$, we get

$$\begin{aligned}
(\gamma_u+\mu)+\tau-(\gamma_u+\mu)R_0^u-(\gamma_u+\tau+\mu)R_0^t &> \tau(R_0^w - R_0^t)\\
\implies (\gamma_u+\mu)+\tau-(\gamma_u+\mu)R_0^u-(\gamma_u+\mu)R_0^t &> \tau R_0^w\\
\implies (\gamma_u+\mu)+\tau-(\gamma_u+\mu)R_0^w &> \tau R_0^w\\
\implies \gamma_u+\mu+\tau &> (\tau+\gamma_u+\mu)R_0^w\\
\implies 1 &> R_0^w
\end{aligned}$$

3.2 RFP

For the RFP

$$\lambda_1^2 = -\frac{\mu}{2}R_0^r - \sqrt{\mu(\gamma_r+\mu)\left(1-R_0^r+\frac{\mu}{4(\gamma_r+\mu)}(R_0^r)^2\right)} \tag{20}$$

$$\lambda_2^2 = -\frac{\mu}{2}R_0^r + \sqrt{\mu(\gamma_r+\mu)\left(1-R_0^r+\frac{\mu}{4(\gamma_r+\mu)}(R_0^r)^2\right)} \tag{21}$$

$$\lambda_2^3 = -\frac{1}{2R_0^r}\left\{(2\gamma_u+\tau+2\mu)R_0^r-(\gamma_u+\tau+\mu)R_0^t-(\gamma_u+\mu)R_0^u\right. \tag{22}$$

$$\left.+\{\sqrt{[\tau R_0^r-(\gamma_u+\tau+\mu)R_0^t]^2+2(\gamma_u+\mu)R_0^u(\tau R_0^r+(\gamma_u+\tau+\mu)R_0^t)+(\gamma_u+\mu)^2(R_0^u)^2}\right\} \tag{23}$$

$$\lambda_2^4 = -\frac{1}{2R_0^r}\left\{(2\gamma_u+\tau+2\mu)R_0^r-(\gamma_u+\tau+\mu)R_0^t-(\gamma_u+\mu)R_0^u\right. \tag{24}$$

$$\left.-\sqrt{[\tau R_0^r-(\gamma_u+\tau+\mu)R_0^t]^2+2(\gamma_u+\mu)R_0^u(\tau R_0^r+(\gamma_u+\tau+\mu)R_0^t)+(\gamma_u+\mu)^2(R_0^u)^2}\right\} \tag{25}$$

For λ_1^2 and λ_2^2 to be a pair of conjugate complex numbers with negative real part, it is necessary that

$$1-R_0^r+\frac{\mu}{4(\mu+\gamma)}(R_0^r)^2<0. \tag{26}$$

Solving for R_0^r gives the condition

$$\frac{2(\gamma+\mu)}{\mu}\left(1-\sqrt{1-\frac{\mu}{\gamma+\mu}}\right) < R_0^r < \frac{2(\gamma+\mu)}{\mu}\left(1+\sqrt{1-\frac{\mu}{\gamma+\mu}}\right). \tag{27}$$

If (27) holds, then the RFP is represented by a stable spiral when projected in the (I_t, I_u) plane. Typically $\mu \ll 1$, thus the conditions above can be approximated to $0 < R_0^r < \frac{4(\gamma+\mu)}{\mu} \gg 1$. This range usually encompasses the range of plausible values of R_0^r. Thus, λ_1^2 and λ_2^2 will most likely be a pair of conjugate complex numbers with negative real part.

The term inside the square root in the conjugate pair λ_2^3, λ_2^4 is always positive, thus $\lambda_2^3, \lambda_2^4 \in \mathbb{R}$. Hence, the stability of the RFP depends on their signs. We now prove that $\lambda_2^3 < 0$ and $\lambda_2^4 < 0$ simultaneously if $R_0^r > R_0^w$. First note that $\lambda_2^3 < 0$ and $\lambda_2^4 < 0$ simultaneously if

$$\begin{aligned}&\left[(2\gamma_u+\tau+2\mu)R_0^r-(\gamma_u+\tau+\mu)R_0^t-(\gamma_u+\mu)R_0^u\right]\\&>\sqrt{[\tau R_0^r-(\gamma_u+\tau+\mu)R_0^t]^2+2(\gamma_u+\mu)R_0^u(\tau R_0^r+(\gamma_u+\tau+\mu)R_0^t)+(\gamma_u+\mu)^2(R_0^u)^2}.\end{aligned}$$

Squaring both sides

$$\begin{aligned}&\left[(2\gamma_u+\tau+2\mu)R_0^r-(\gamma_u+\tau+\mu)R_0^t-(\gamma_u+\mu)R_0^u\right]^2\\&>[\tau R_0^r-(\gamma_u+\tau+\mu)R_0^t]^2+2(\gamma_u+\mu)R_0^u(\tau R_0^r+(\gamma_u+\tau+\mu)R_0^t)+(\gamma_u+\mu)^2(R_0^u)^2.\end{aligned}$$

Expanding the left-hand-side yields

$$[(2\gamma_u+\tau+2\mu)R_0^r]^2+[(\gamma_u+\tau+\mu)R_0^t]^2+[(\gamma_u+\mu)R_0^u]^2-$$

$$-2\left\{[(2\gamma_u+\tau+2\mu)R_0^r(\gamma_u+\tau+\mu)R_0^t]+[(2\gamma_u+\tau+2\mu)R_0^r(\gamma_u+\mu)R_0^u]-[(\gamma_u+\tau+\mu)R_0^t(\gamma_u+\mu)R_0^u]\right\}>$$

$$>[\tau R_0^r-(\gamma_u+\tau+\mu)R_0^t]^2+2(\gamma_u+\mu)R_0^u[\tau R_0^r+(\gamma_u+\tau+\mu)R_0^t]+[(\gamma_u+\mu)R_0^u]^2.$$

Canceling $[(\gamma_u+\mu)R_0^u]^2$ and expanding $[\tau R_0^r-(\gamma_u+\tau+\mu)R_0^t]^2$ yields

$$[(2\gamma_u+\tau+2\mu)R_0^r]^2+[(\gamma_u+\tau+\mu)R_0^t]^2-$$

$$-2\left\{[(2\gamma_u+\tau+2\mu)R_0^r(\gamma_u+\tau+\mu)R_0^t]+[(2\gamma_u+\tau+2\mu)R_0^r(\gamma_u+\mu)R_0^u]-[(\gamma_u+\tau+\mu)R_0^t(\gamma_u+\mu)R_0^u]\right\}>$$

$$>[\tau R_0^r]^2+[(\gamma_u+\tau+\mu)R_0^t]^2-2[(\gamma_u+\tau+\mu)R_0^t][\tau R_0^r]+2(\gamma_u+\mu)R_0^u\tau R_0^r+2(\gamma_u+\mu)R_0^u(\gamma_u+\tau+\mu)R_0^t].$$

Canceling $[(\gamma_u+\tau+\mu)R_0^t]^2$ one obtains

$$[(2\gamma_u+\tau+2\mu)R_0^r]^2-$$

$$-2\left\{[(2\gamma_u+\tau+2\mu)R_0^r(\gamma_u+\tau+\mu)R_0^t]+[(2\gamma_u+\tau+2\mu)R_0^r(\gamma_u+\mu)R_0^u]-[(\gamma_u+\tau+\mu)R_0^t(\gamma_u+\mu)R_0^u]\right\}>$$

$$>[\tau R_0^r]^2-2[(\gamma_u+\tau+\mu)R_0^t][\tau R_0^r]+2(\gamma_u+\mu)R_0^u\tau R_0^r+2(\gamma_u+\mu)R_0^u(\gamma_u+\tau+\mu)R_0^t].$$

Canceling $2[(\gamma_u+\tau+\mu)R_0^t(\gamma_u+\mu)R_0^u]$ yields

$$[(2\gamma_u+\tau+2\mu)R_0^r]^2-2\left\{[(2\gamma_u+\tau+2\mu)R_0^r(\gamma_u+\tau+\mu)R_0^t]+[(2\gamma_u+\tau+2\mu)R_0^r(\gamma_u+\mu)R_0^u]\right\}>$$

$$>[\tau R_0^r]^2-2[(\gamma_u+\tau+\mu)R_0^t][\tau R_0^r]+2(\gamma_u+\mu)R_0^u\tau R_0^r.$$

Dividing both sides by R_0^r renders

$$R_0^r[(2\gamma_u+\tau+2\mu)]^2-2[(2\gamma_u+\tau+2\mu)(\gamma_u+\tau+\mu)R_0^t]-2[(2\gamma_u+\tau+2\mu)(\gamma_u+\mu)R_0^u]>$$

$$>\tau^2R_0^r-2[(\gamma_u+\tau+\mu)R_0^t]\tau+2(\gamma_u+\mu)R_0^u\tau.$$

Factoring R_0 terms we get

$$-2[(2\gamma_u+\tau+2\mu)(\gamma_u+\tau+\mu)-(\gamma_u+\tau+\mu)\tau]R_0^t-2[(2\gamma_u+\tau+2\mu)(\gamma_u+\mu)+\tau(\gamma_u+\mu)]R_0^u>$$

$$>[\tau^2-[(2\gamma_u+\tau+2\mu)]^2]R_0^r.$$

Expanding and canceling τ terms yields

$$-2[(2\gamma_u+2\mu)(\gamma_u+\tau+\mu)]R_0^t-2[(2\gamma_u+2\tau+2\mu)(\gamma_u+\mu)]R_0^u>[-(2\gamma_u+2\mu)^2-2(2\gamma_u+2\mu)\tau]R_0^r.$$

Dividing both sides by 4 yields

$$-[(\gamma_u+\mu)(\gamma_u+\tau+\mu)]R_0^t - [(\gamma_u+\tau+\mu)(\gamma_u+\mu)]R_0^u > [-(\gamma_u+\mu)^2 - (\gamma_u+\mu)\tau]R_0^r.$$

Dividing both sides by $(\gamma_u+\mu)$ renders

$$(\gamma_u+\tau+\mu)R_0^t + (\gamma_u+\mu+\tau)R_0^u < (\gamma_u+\mu+\tau)R_0^r.$$

Dividing both sides by $(\gamma_u+\tau+\mu)$ we finally get

$$R_0^w < R_0^r.$$

3.3 Effect of Varying ρ and ϕ: Phase Planes

In the figures below the following parameters have been fixed: $c = \frac{1}{500}$, $\mu = 4.57\cdot 10^{-5}$, $\gamma = \frac{1}{5}$, $\beta_u = \frac{1}{2}$, $m = 0.34$. The death rate expresses a mean life expectancy of 60 years. β_u and γ yield $R_0 = 2.5$.

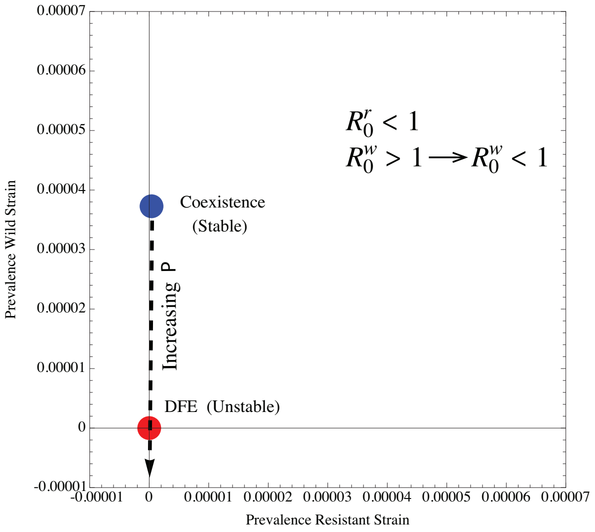

Figure S4. Transcritical Bifurcation between DFE and CFP.

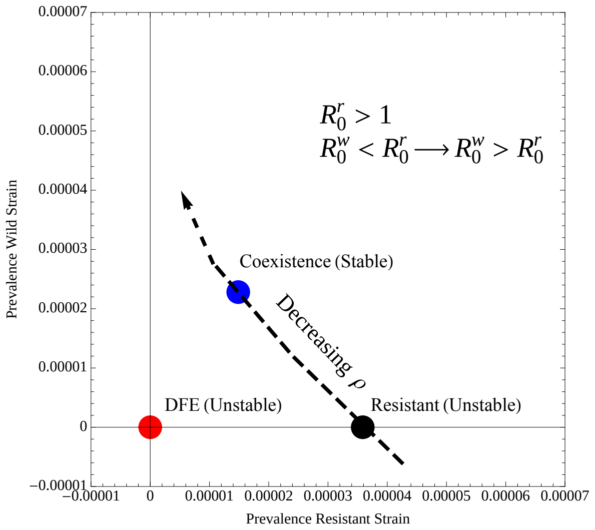

Figure S5. Transcritical Bifurcation between RFP and CFP.

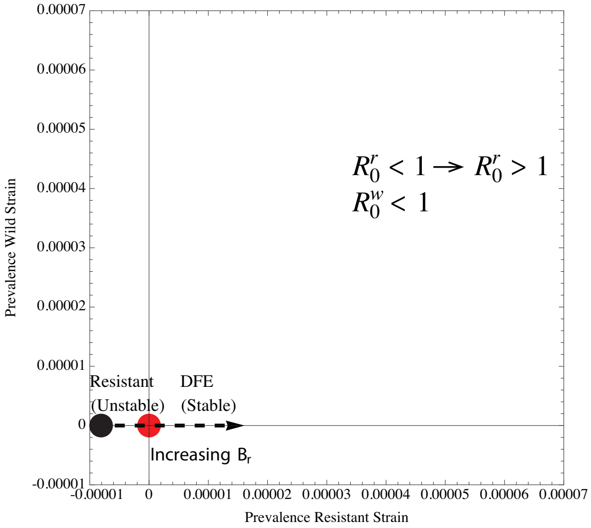

Figure S6. Transcritical Bifurcation between the DFE and the RFP.

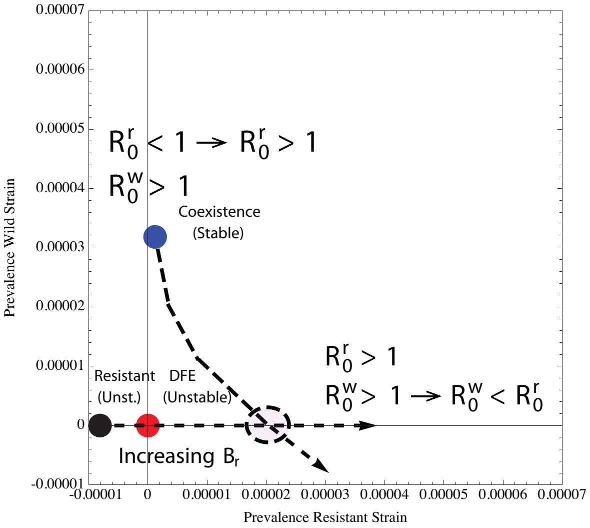

Figure S7. Transcritical Bifurcation between RFP and CFP.

3.4 Summary of the Stability Behavior of the System

Figure S8 summarizes the stability behavior of the system as a function of R_0^w and R_0^r, and shows which key parameters need to change to shift from one stability region to another.

Going clockwise, starting in the top-left corner with high ρ and low ϕ, the DFE is stable. As ρ decreases, R_0^w surpasses the threshold of 1 and the system enters the Coexistence 2FP region (CFP stable, DFE unstable). It is easy to show that if $R_0^w = 1$, then DFE = CFP. Since the FPs also exchange their stability at this point, this represents a transcritical bifurcation [2]. Incrementing ϕ above ϕ^1, R_0^r crosses the threshold of 1, and shifts the system to the 3FP region (CFP stable, DFE unstable, RFP unstable). Further increasing ϕ (increasing R_0^r beyond R_0^w) or increasing $\rho(> \rho^*)$ (reducing R_0^w bellow R_0^r), the system enters the Resistance region (DFE unstable, RFP stable). If $R_0^w = R_0^r$, then CFP=RFP, which implies that the CFP exits the BS area crossing through the RFP. Also here, the FPs exchange stability, featuring a transcritical bifurcation. If $\rho > \rho^1$ and $\phi < \phi^1$, the system goes from the Resistant to the DFE region. If $R_0^r = 1$, then DFE = RFP, and they exchange stability, displaying once more a transcritical bifurcation.

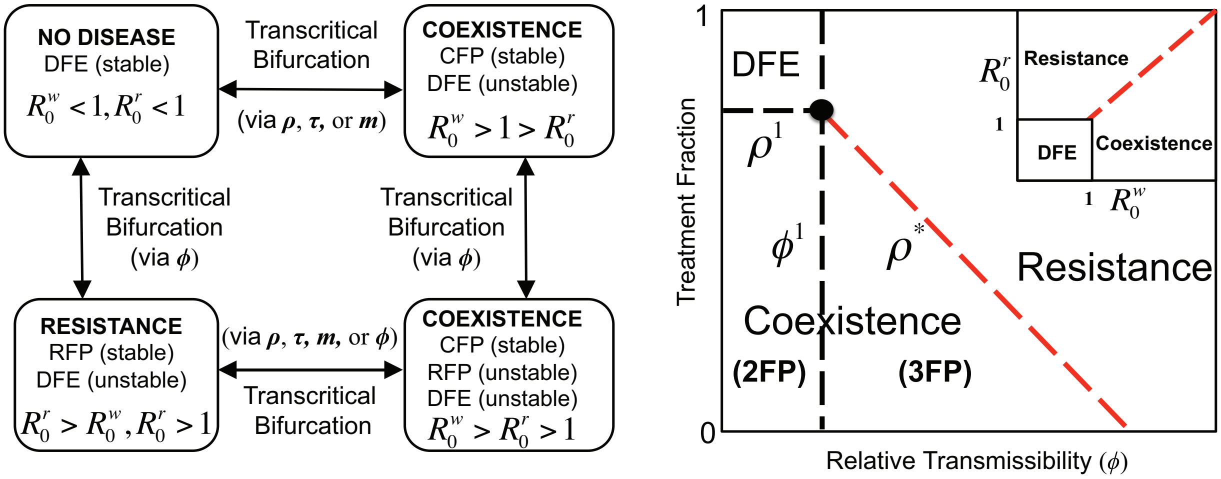

Figure S8. Stability behavior of the system. Depending on the values of R_0^w and R_0^r, the system transits through different stability regions by varying specific parameters.

3.5 Prevalence as a Function of ρ and ϕ

To have a broader idea of what effect the resistant strain fitness has on the overall disease prevalence, Figure S9 shows a plot of the prevalence as a function of ρ and ϕ. For comparative purposes, the red-dashed line coincides with the red-dashed line in Figure S8 (right), and the red and black solid line represent the steady state trajectories in the Figure 4 in the main text. For fixed ϕ, increasing ρ has an effect on the prevalence only up to the red-dashed line ($\rho = \rho^*$). Conversely, for fixed ρ, increasing ϕ has no effect on prevalence if $R_0^w(\rho) > R_0^r(\phi)$. After that threshold (red-dashed line), increasing ϕ also increases prevalence of the resistant strain.

4 Finding the Optimal Treatment Regimes

The FP that dictate endemic levels of disease are :

RFP:

$$S^2 = \frac{1}{R_0^r}, \quad I_t^2 = 0, \quad I_u^2 = 0, \quad I_r^2 = \frac{\mu}{\phi \beta_u}(R_0^r - 1) \tag{28}$$

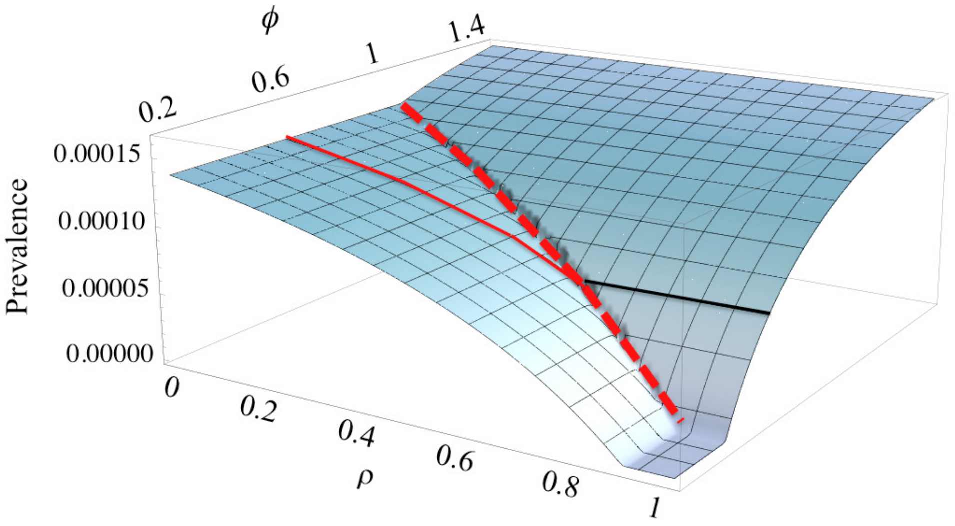

Figure S9. Prevalence as a function of ρ and ϕ with $c = 1/500$.

CFP:

$$S^3 = \frac{1}{R_0^w}, \quad I_t^3 = \frac{(1-c)\rho\mu}{(\gamma_u + \tau + \mu)}\left(1 - \frac{R_0^r}{R_0^w}\right)\Psi, \quad I_u^3 \;= \frac{(1-\rho)\mu}{(\gamma_u + \mu)}\left(1 - \frac{R_0^r}{R_0^w}\right)\Psi, \quad I_r^3 = c\rho\frac{\mu}{(\gamma_r + \mu)}\Psi \tag{29}$$

where

$$\Psi := \frac{R_0^w - 1}{R_0^w - (1 - c\rho)R_0^r}.$$

Clearly, the RFP does not depend on the treatment level ρ, while CFP does.

4.1 Overall fitness and the role of c

In this analysis, lets assume for simplicity that $\tau = 0$ and $\gamma_u = \gamma_r$, yielding $\sigma_i = \sigma, i \in \{u, t, r\}$. From Eq. (4) in the model, we get for the resistant strain

$$\frac{dI_r}{dt} = \theta_r S + \theta_w S\rho c - \sigma I_r = \left(\frac{\theta_r}{I_r\sigma}S + \frac{\theta_w\rho c}{I_r\sigma}S - 1\right)\sigma I_r = \left(R_0^r S + \frac{\theta_w\rho c}{I_r\sigma}S - 1\right)\sigma I_r$$

Using the approximation

$$\frac{dI_r}{dt} \approx \frac{I_r^{n+1} - I_r^n}{1/\sigma},$$

where $1/\sigma$ is the expected duration of an "epidemic generation" and n indexes the generations, renders

$$I_r^{n+1} \approx \left(R_0^r S + \frac{\theta_w \rho c}{I_r \sigma} S \right) I_r^n.$$

Assuming a susceptible-rich population, we can approximate $S \approx 1$, finally yielding

$$I_r^{n+1} \approx \left(R_0^r + \frac{\theta_w \rho c}{I_r^n \sigma} \right) I_r^n = R_0^r I_r^n + \frac{\theta_w \rho c}{\sigma} \tag{30}$$

Proceeding similarly for the wild-type strain we get

$$\frac{dI_t}{dt} = \theta_w S \rho (1-c) - \sigma I_t \implies I_t^{n+1} \approx \left(\rho(1-c) \frac{\theta_w}{I_t^n \sigma} \right) I_t^n$$
$$\frac{dI_u}{dt} = \theta_w S (1-\rho) - \sigma I_u \implies I_u^{n+1} \approx \left((1-\rho) \frac{\theta_w}{I_u^n \sigma} \right) I_u^n$$

Let $I_w^{n+1} = I_u^n + I_t^n$, then

$$\begin{aligned} I_w^{n+1} \approx \left(\rho(1-c) \frac{\theta_w}{\sigma} \right) + \left((1-\rho) \frac{\theta_w}{\sigma} \right) &= \left((1-\rho c) \frac{\theta_w}{I_w^n \sigma} \right) I_w^n \\ &= \left((1-\rho c) \frac{\beta_u I_u^n + m \beta_u I_t^n}{(I_u^n + I_t^n)\sigma} \right) I_w^n \\ &= (1-\rho c) \left(\frac{\beta_u i_u^n + m \beta_u i_t^n}{\sigma} \right) I_w^n \end{aligned}$$

where i_x^n is the fraction of the wild type infected that did not develop resistance and ended up in class $x \in \{u, t\}$ in generation n. It follows then that $i_u^n = \dfrac{(1-\rho)}{1-\rho c}$ and $i_t^n = \dfrac{\rho(1-c)}{1-\rho c}$. Thus

$$I_w^{n+1} \approx (1-\rho c) \left(\frac{\beta_u \frac{(1-\rho)}{1-\rho c} + m \beta_u \frac{\rho(1-c)}{1-\rho c}}{\sigma} \right) I_w^n = R_0^w I_w^n \tag{31}$$

Since $(1-\rho c) \dfrac{\theta_w}{I_w^n \sigma} = R_0^w$, from Eq. (30) we then get

$$I_r^{n+1} \approx R_0^r I_r^n + \frac{\rho c}{1-\rho c} R_0^w I_w^n \tag{32}$$

The absolute fitness of strain $k \in \{w, r\}$ can be defined as $F_k = \dfrac{I_k^{n+1}}{I_k^n}$, that is, how many new infections

("offspring") did each infected contribute to the next generation on average. For the wild-type strain this definition holds. However, for the resistant strain the *de novo* resistant cases are not "produced" by resistant strain infections, so the definition does not hold for the *de novo* contribution term. Instead, the *de novo* term should be divided by I_w^n. Then, from Eq. (31) and Eq. (32) we obtain

$$F_w = R_0^w H(\rho^* - \rho) \text{ and } F_r = R_0^r + \frac{\rho c}{1-\rho c} R_0^w H(\rho^* - \rho).$$

where $H(x)$ is the Heaviside step function: $H(x) = 1$ if $x > 0$, and $H(x) = 0$ otherwise. It is used to signify that if $\rho > \rho^*$, there are no more wild-type cases, and therefore, no *de novo* cases either. The *de novo* term can be interpreted as the number of new *de novo* infections that each wild-type infected legated to the next generation, in average. Note that as $c \to 0$, then $F_w \to R_0^w$ and $F_r \to R_0^r$.

4.2 Exploring the monotonicity of $I_r^3(\rho)$ in $(0, \rho^*)$

From the expression of I_r^3 it is clear that

$$\frac{\partial I_r^3}{\partial \rho} = \frac{c\mu}{\gamma_r + \mu}\Psi + \frac{c\mu\rho}{\gamma_r + \mu}\frac{\partial \Psi}{\partial \rho} = \frac{c\mu}{\gamma_r + \mu}\left(\Psi + \rho\frac{\partial \Psi}{\partial \rho}\right)$$

Thus, for $\frac{\partial I_r^3}{\partial \rho} > 0$ we must show that $\Psi + \rho\frac{\partial \Psi}{\partial \rho} > 0$, where

$$\begin{aligned}
\Psi + \rho\frac{\partial \Psi}{\partial \rho} &= \frac{R_0^w - 1}{R_0^w - (1-c\rho)R_0^r} + \rho\frac{\frac{\partial R_0^w}{\partial \rho}\left(1 - (1-c\rho)\, R_0^r\right) - (R_0^w - 1)cR_0^r}{\left(R_0^w - (1-c\rho)R_0^r\right)^2} \\
&= \frac{(R_0^w - 1)(R_0^w - (1-c\rho)R_0^r) + \rho\frac{\partial R_0^w}{\partial \rho}\left(1 - (1-c\rho)\, R_0^r\right) - (R_0^w - 1)c\rho R_0^r}{(R_0^w - (1-c\rho)R_0^r)^2} \qquad (33)
\end{aligned}$$

Since the denominator of this last expression is always positive, we focus only on the sign of the numerator

$$
\begin{aligned}
&(R_0^w - 1)(R_0^w - (1 - c\rho)R_0^r) + \rho\frac{\partial R_0^w}{\partial\rho}(1 - (1 - c\rho)R_0^r) - (R_0^w - 1)c\rho R_0^r \\
=&(R_0^w - 1)R_0^w - (R_0^w - 1)(1 - c\rho)R_0^r + \rho\frac{\partial R_0^w}{\partial\rho}(1 - (1 - c\rho)R_0^r) - (R_0^w - 1)c\rho R_0^r \\
=&(R_0^w - 1)R_0^w - (R_0^w - 1)R_0^r + \rho\frac{\partial R_0^w}{\partial\rho}(1 - (1 - c\rho)R_0^r) \\
=&(R_0^w - 1)(R_0^w - R_0^r) + \rho\frac{\partial R_0^w}{\partial\rho}(1 - (1 - c\rho)R_0^r) \qquad (34)
\end{aligned}
$$

From (33) notice that

$$
1 - (1 - c\rho)R_0^r \leq 0 \implies R_0^r \geq \frac{1}{1 - \rho c}. \qquad (35)
$$

If the inequality in (35) holds, and recalling that $R_0^w > 1$, $R_0^w > R_0^r$ and $\frac{\partial R_0^w}{\partial\rho} < 0$, for $\rho < \rho^*$, we have found sufficient conditions to show that $\frac{\partial I_r^3}{\partial\rho} > 0$.

Since we are working on the range $0 \leq \rho < \rho^*$, we get that $\frac{1}{1 - \rho c} < \frac{1}{1 - \rho^* c}$. Thus, if the inequality in (35) does not hold (i.e., $1 \leq R_0^r < \frac{1}{1 - \rho^* c}$)[1], we cannot assure that I_r^3 is a monotonically increasing function of ρ.

To approach the question of whether $\frac{\partial I_r^3}{\partial\rho} > 0$ or not if $1 \leq R_0^r < \frac{1}{1 - \rho^* c}$ given $\rho \in [0, \rho^*]$, we proceed as follows. Showing that $\left.\frac{\partial I_r^3}{\partial\rho}\right|_{\rho=\rho^*} < 0$ is a sufficient condition to prove that I_r^3 is *not* a monotonically increasing function of ρ in the interval $\rho \in [0, \rho^*]$. Recalling that $R_0^w(\rho^*) = R_0^r$, expression (34) becomes

$$
\rho^* \frac{\partial R_0^w}{\partial\rho}(1 - (1 - c\rho^*)R_0^r).
$$

Then, it is easy to see that if $R_0^r < \frac{1}{1 - \rho^* c}$, then the above expression is negative. Thus, we have shown that in this case, I_r^3 is *not* a monotonically increasing function of ρ in the interval $\rho \in [0, \rho^*]$. Moreover, numerically we find that in such case $I_r^3(\rho)$ is a concave function in the interval $[0, \rho^*]$ (see Figure S10). This behavior is more accentuated for larger c.

[1]Since $\rho \in [0, \rho^*]$, then $(1 - \rho c)^{-1} < (1 - \rho^* c)^{-1}$. Also, the interesting cases are those in which the resistant strain can potentially emerge and persist, i.e., $I_r^2 > 0$. Thus, $R_0^r \geq 1$.

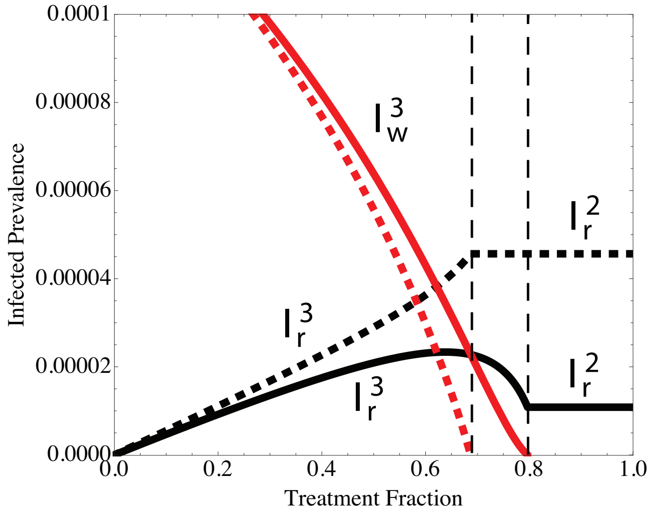

Figure S10. $I_r^3(\rho)$ for $1 < R_0^r < \dfrac{1}{1-\rho^* c}$ (black, solid) ($\phi = 0.42$) and for $R_0^r > \dfrac{1}{1-\rho^* c}$ (red, solid) ($\phi = 0.5$). Dotted lines are the corresponding $I_w^3(\rho)$ curves. The vertical dashed lines are the values of ρ^* corresponding to $\phi = 0.42, 0.5$. If we are in the first case, then increasing treatment is the best. Other parameters: $\gamma_r = \gamma_u = 0.2, \tau = 0, \beta_u = 0.5, m = 0.34, c = 0.2$. A low value of c was used to magnify the difference between the two cases.

4.3 Showing that $\dfrac{\partial I_w^3}{\partial \rho} < 0$ for $0 < \rho < \rho^*$

Let $I_w^3 = I_u^3 + I_t^3$ and $\xi := 1 - \dfrac{R_0^r}{R_0^w}$, then

$$I_w^3 = \xi \Psi \left[\rho \left(\frac{(1-c)\mu}{\sigma_t} - \frac{\mu}{\sigma_u} \right) + \frac{\mu}{\sigma_u} \right]. \tag{36}$$

Therefore

$$\frac{\partial I_w^3}{\partial \rho} = \left(\frac{\partial \xi}{\partial \rho} \Psi + \xi \frac{\partial \Psi}{\partial \rho} \right) \left[\rho \left(\frac{(1-c)\mu}{\sigma_t} - \frac{\mu}{\sigma_u} \right) + \frac{\mu}{\sigma_u} \right] + \xi \Psi \left(\frac{(1-c)\mu}{\sigma_t} - \frac{\mu}{\sigma_u} \right) \tag{37}$$

Notice that since $\Psi > 0$ and $\xi > 0$ in this epidemiological context, the second term in (37) is always negative (recall $\sigma_u < \sigma_t$). Expanding the term in brackets in (37) we get

$$\rho \left(\frac{(1-c)\mu}{\sigma_t} - \frac{\mu}{\sigma_u} \right) + \frac{\mu}{\sigma_u} = \mu \left(\frac{\rho(1-c)\sigma_u - \rho\sigma_t + \sigma_t}{\sigma_t \sigma_u} \right) = \mu \left(\frac{\rho(1-c)\sigma_u + (1-\rho)\sigma_t}{\sigma_t \sigma_u} \right) > 0$$

Thus, from (37) it is clear that to prove $\dfrac{\partial I_w^3}{\partial \rho} < 0$ we need to prove that $\left(\dfrac{\partial \xi}{\partial \rho} \Psi + \xi \dfrac{\partial \Psi}{\partial \rho} \right) < 0$. We have

$$\frac{\partial \Psi}{\partial \rho} = \frac{R_0^r}{(R_0^w)^2}\frac{\partial R_0^w}{\partial \rho} \quad \text{and} \quad \frac{\partial \xi}{\partial \rho} = \frac{\frac{\partial R_0^w}{\partial \rho}[1-(1-\rho c)R_0^r] - cR_0^r(R_0^w-1)}{[R_0^w-(1-\rho c)R_0^r]^2}$$

Thus, $\left(\frac{\partial \xi}{\partial \rho}\Psi + \xi\frac{\partial \Psi}{\partial \rho}\right)$ becomes

$$\frac{\left[\frac{\partial R_0^w}{\partial \rho}[1-(1-\rho c)R_0^r] - cR_0^r(R_0^w-1)\right]\left[(R_0^w)^2 - R_0^w R_0^r\right] + R_0^r\frac{\partial R_0^w}{\partial \rho}(R_0^w-1)[R_0^w-(1-\rho c)R_0^r]}{(R_0^w)^2[R_0^w-(1-\rho c)R_0^r]^2} \tag{38}$$

Recognizing that the denominator of (38) is always positive, we focus on the sign of the numerator. The second term in the sum of the numerator is always negative given that $\frac{\partial R_0^w}{\partial \rho} < 0$. In the first term, the term in the second square brackets is always positive. Conversely, the term in the first square brackets is negative if $1-(1-\rho c)R_0^r > 0$. If this last inequality holds, then we have found sufficient conditions to prove that $\left(\frac{\partial \xi}{\partial \rho}\Psi + \xi\frac{\partial \Psi}{\partial \rho}\right) < 0$ and consequently that $\frac{\partial I_r^3}{\partial \rho} > 0$ in $(0, \rho^*)$.

We now use heuristic arguments to show $\frac{\partial I_w^3}{\partial \rho} < 0$ for the case $1 < (1-\rho c)R_0^r$. For this case we already showed analytically that $\frac{\partial I_r^3}{\partial \rho} > 0$ for $\rho \in [0, \rho^*]$. We interpret these partial derivatives as flows *from* and *to* the infected and the susceptible classes. For instance, $F_S = \frac{S(\rho) - S(\rho+\delta\rho)}{\delta\rho}$ is the flow of individuals to the S class due to a change in treatment $\delta\rho$. Based on a conservation of mass (individuals) argument, we can write

$$F_{S^3} + F_{I_w^3} + F_{I_r^3} \equiv 0. \tag{39}$$

Notice that, unlike the unidirectional flow of individuals in time (measured by the time derivatives), the flow with respect to ρ allows individuals to move back and forth within these classes. If $1 < (1-\rho c)R_0^r$, then $F_{I_r^3}$ is positive. It is also easy to check that if $\frac{\partial R_0^w}{\partial \rho} < 0$, then $F_{S^3} \approx \frac{\partial S_3}{\partial \rho} = -\frac{1}{(R_0^w)^2}\frac{\partial R_0^w}{\partial \rho} > 0$. Hence, increasing ρ increases the flow of individuals towards S^3 and I_r^3; thus to satisfy expression (39) we must have $F_{I_w^3} \approx \frac{\partial I_w^3}{\partial \rho} < 0$ for $\rho \in [0, \rho^*]$. This proves that $\frac{\partial I_w^3}{\partial \rho} < 0$ also for the case $1 < (1-\rho c)R_0^r$, and since we had already proved it when $1 > (1-\rho c)R_0^r$, we have shown that indeed $\frac{\partial I_w^3}{\partial \rho} < 0$ for $\rho \in [0, \rho^*]$.

4.4 Finding ρ_e and ρ_r

We obtain ρ_e from $I_w^3(\rho_e) = I_r^3(\rho_e)$. This yields

$$\rho_e = \frac{1}{2\alpha_1\alpha_2}\left\{\sigma_t^2(2\sigma_r + \sigma_u(c-\phi)) - (1-c)\sigma_t\sigma_u(\sigma_r - \sigma_u\phi + \sigma_r m) - \right.$$

$$\left.\sqrt{\sigma_t^2\{-4\alpha_1(\sigma_r-\sigma_u\phi)\alpha_2+[c\sigma_t\sigma_u-\sigma_u(\sigma_t-(1-c)\sigma_u)\phi+\sigma_r[2\sigma_t-(1-c)\sigma_u(1+m)]]^2\}}\right\}$$

where we have defined $\sigma_t = \gamma_u + \tau + \mu, \sigma_u = \gamma_u + \mu$ and $\sigma_r = \gamma_r + \mu$. Also $\alpha_1 = c\sigma_t\sigma_u + \sigma_r(\sigma_t - (1-c)\sigma_u)$ and $\alpha_2 = \sigma_t - (1-c)\sigma_u m$.

We derive ρ_r from $I_r^3 = I_r^2$, yielding

$$\begin{aligned}
I_r^3(\rho) &= I_r^2 \\
c\rho\frac{\mu}{\sigma_r}\frac{R_0^w-1}{R_0^w-(1-c\rho)R_0^r} &= \frac{\mu(R_0^r-1)}{\phi\beta_u} \\
\frac{c\rho}{R_0^w-(1-c\rho)R_0^r} &= \frac{(R_0^r-1)}{R_0^r(R_0^w-1)} \\
c\rho &= \frac{(R_0^r-1)}{R_0^r(R_0^w-1)}(R_0^w-(1-c\rho)R_0^r) \\
c\rho &= \frac{(R_0^r-1)(R_0^w-R_0^r)}{R_0^r(R_0^w-1)} + \frac{(R_0^r-1)}{R_0^r(R_0^w-1)}c\rho R_0^r \\
c\rho\left[1-\frac{(R_0^r-1)}{R_0^r(R_0^w-1)}R_0^r\right] &= \frac{(R_0^r-1)(R_0^w-R_0^r)}{R_0^r(R_0^w-1)} \\
c\rho\left[\frac{R_0^r(R_0^w-1)-(R_0^r-1)R_0^r}{R_0^r(R_0^w-1)}\right] &= \frac{(R_0^r-1)(R_0^w-R_0^r)}{R_0^r(R_0^w-1)} \\
c\rho\left[R_0^r(R_0^w-1)-(R_0^r-1)R_0^r\right] &= (R_0^r-1)(R_0^w-R_0^r) \\
c\rho\left[R_0^rR_0^w-(R_0^r)^2\right] &= (R_0^r-1)(R_0^w-R_0^r) \\
\implies \rho_r &= \frac{R_0^r-1}{R_0^r c}.
\end{aligned}$$

References

1. van den Driessche P, Watmough J (2002) Reproduction numbers and sub-threshold endemic equilibria for compartmental models of disease transmission. Math Biosci 180: 29-48.

2. Strogatz SH (1994) Nonlinear dynamics and Chaos: with applications to physics, biology, chemistry, and engineering. Addison-Wesley Pub.
